# A new link between transcriptional initiation and pre-mRNA splicing: The RNA binding histone variant H2A.B

**DOI:** 10.1371/journal.pgen.1006633

**Published:** 2017-02-24

**Authors:** Tatiana A. Soboleva, Brian J. Parker, Maxim Nekrasov, Gene Hart-Smith, Ying Jin Tay, Wei-Quan Tng, Marc Wilkins, Daniel Ryan, David J. Tremethick

**Affiliations:** 1 The John Curtin School of Medical Research, The Australian National University, Canberra, Australia; 2 NSW Systems Biology Initiative, University of New South Wales, Sydney, Australia; IGBMC, FRANCE

## Abstract

The replacement of histone H2A with its variant forms is critical for regulating all aspects of genome organisation and function. The histone variant H2A.B appeared late in evolution and is most highly expressed in the testis followed by the brain in mammals. This raises the question of what new function(s) H2A.B might impart to chromatin in these important tissues. We have immunoprecipitated the mouse orthologue of H2A.B, H2A.B.3 (H2A.Lap1), from testis chromatin and found this variant to be associated with RNA processing factors and RNA Polymerase (Pol) II. Most interestingly, many of these interactions with H2A.B.3 (Sf3b155, Spt6, DDX39A and RNA Pol II) were inhibited by the presence of endogenous RNA. This histone variant can bind to RNA directly *in vitro* and *in vivo*, and associates with mRNA at intron—exon boundaries. This suggests that the ability of H2A.B to bind to RNA negatively regulates its capacity to bind to these factors (Sf3b155, Spt6, DDX39A and RNA Pol II). Unexpectedly, H2A.B.3 forms highly decompacted nuclear subdomains of active chromatin that co-localizes with splicing speckles in male germ cells. H2A.B.3 ChIP-Seq experiments revealed a unique chromatin organization at active genes being not only enriched at the transcription start site (TSS), but also at the beginning of the gene body (but being excluded from the +1 nucleosome) compared to the end of the gene. We also uncover a general histone variant replacement process whereby H2A.B.3 replaces H2A.Z at intron-exon boundaries in the testis and the brain, which positively correlates with expression and exon inclusion. Taken together, we propose that a special mechanism of splicing may occur in the testis and brain whereby H2A.B.3 recruits RNA processing factors from splicing speckles to active genes following its replacement of H2A.Z.

## Author summary

The substitution of core histones with their non-allelic variant forms plays a particular important role in regulating chromatin function because they can directly alter the structure of chromatin, and provide new protein interaction interfaces for the recruitment of proteins involved in gene expression. Despite being discovered over a decade ago, the function of H2A.B, a variant of the H2A class, in its proper physiological context (being expressed in the testis and the brain) is unknown. We provide strong evidence that H2A.B has a role in the processing of RNA. It is found in the gene body of an active gene, directly interacts with RNA polymerase II and splicing factors and is located in the nucleus at distinct regions enriched with RNA processing factors (splicing speckles). Most significantly, we show that H2A.B can directly bind to RNA both *in vitro* and in germ cells. Therefore, H2A.B has the novel ability to bind to both RNA and DNA (as well as proteins) thus directly linking chromatin structure with the function of RNA. Taken together, this suggests that a special mechanism of splicing may operate in the testis and brain.

## Introduction

Histones, the key proteins that compact all eukaryotic DNA into chromatin, have attracted much attention recently because of their impact on all aspects of genome function [1]. Histones form the core structure of chromatin, the nucleosome core, in which ~ 145 base pairs of DNA is wrapped around a histone octamer comprising of a (H3-H4)_2_ tetramer flanked by two H2A-H2B dimers. Importantly, the structure and function of a nucleosome can be regulated by the substitution of one or more of the major core histones with their variant forms.

Despite being discovered by Chadwick and colleagues over a decade ago, the function of the histone H2A variant, H2A.Bbd remains unknown [2]. *In vitro* biophysical and transcription studies revealed that H2A.Bbd, and the mouse orthologue, which we designated H2A.Lap1 (Lack of an acidic patch) [3], could not compact chromatin *in vitro* [4]. Functionally, this permitted high levels of RNA polymerase (Pol) II transcription [4]. Adopting the new nomenclature for histone variants [5], H2A.Bbd and H2A.Lap1 will hereafter be referred to as H2A.B and H2A.B.3, respectively.

H2A.B histones differ from their canonical counterparts in several important ways. First, H2A.B histones have a reduced acidic patch, a key region on the nucleosome surface required for chromatin compaction [3]. Second, H2A.B histones lack the canonical histone carboxyl-terminal region, which is important for stabilizing the interaction interface between the H3–H4 tetramer and the H2A—H2B dimer [6]. Not surprisingly, H2A.B-containing mononucleosomes are unstable [7,8], which is also consistent with numerous studies showing the unwrapping of nucleosomal DNA from the octamer surface at the DNA entry and exit points [8–10]. This unwrapping of DNA also appears to cause a major reorganization of the histone tails within the nucleosome [11], and allows octamer formation on DNA fragments smaller than the typical 145 base pairs *in vitro* that are associated with a canonical nucleosome [12]. Third, the N-terminal tails of H2A.B histones distinctively lack lysine residues, but instead are enriched for arginines. The functional significance of this difference is as yet unknown.

H2A.B is a rapidly evolving histone variant family that first appeared in mammals [5,13]. Notably, it displays a tissue-restricted expression pattern being highly expressed in the adult testis with some expression in the brain, and is encoded by three genes [3] (NCBI GEO data sets). It also appears to be expressed in mouse embryonic stem cells at a low level [14]. Several studies have overexpressed tagged versions of H2A.B in transformed cell lines [2,15,16] and following genome-wide analyses revealed it to be preferentially associated with actively transcribed genes. Over expression of H2A.B can have abnormal effects on cell cycle regulation and DNA damage [16].

To begin to understand the possible role(s) of H2A.B and its orthologues in its proper physiological context, we previously performed H2A.B.3 immunofluorescence, ChIP-Seq and expression studies in the mouse testis [3]. A new role for H2A histone variants in the transcriptional activation process was uncovered whereby H2A.B.3 was specifically targeted to the transcription start site (TSS) of active genes, which was previously believed to be nucleosome free [3,17]. This location was not observed in the above mentioned H2A.B studies in transformed cell lines. We now show here that H2A.B.3 is also present at the TSS of active genes in the mouse brain.

In order to gain new mechanistic insights into how H2A.B.3 participates in the gene activation process, here we took a proteomic approach to identify proteins that specifically interact with H2A.B-containing nucleosomes in the mouse testis, analysed its pattern of organisation in germ cell nuclei, uncovered new genomic locations for this histone variant, examined its functional relationship with another histone variant, H2A.Z, and the active H3K36me3 mark, both in the testis and the brain and finally, analysed its interaction with RNA both *in vivo* and *in vitro*.

## Results

### H2A.B.3 is found in the gene body of active genes in the testis

Previously, we identified the mouse orthologue of H2A.B, H2A.B.3, and showed that it is expressed between the pachytene stage (meiosis I, day 19 of spermatogenesis) and the late round spermatid stage (immediately following the completion of meiosis II, day 28–30) [3]. The expression of H2A.B.3 peaks at the late round spermatid stage (its expression is ~8-fold higher at this stage compared to the pachytene stage). This is the period of spermatogenesis when the overall level of transcription is extremely high [18]. Our initial transcriptomic and ChIP-Seq analysis revealed that H2A.B.3 is targeted to the TSS concurrent with gene activation indicating a role in transcription initiation [3]. However, it was unclear whether H2A.B.3 was also located in the body of an active gene, which would indicate other possible functions for this variant in the expression of a gene. To investigate this possibility, we repeated H2A.B.3 ChIP-Seq (at a greater sequencing depth) and RNA-Seq experiments using micrococcal nuclease prepared mononucleosomes and poly (A)-transcripts obtained from 28–30 day old mice testes, respectively. Resulting ChIP-Seq and RNA-Seq libraries were sequenced yielding 100 base pair paired-end reads.

**Fig 1:**
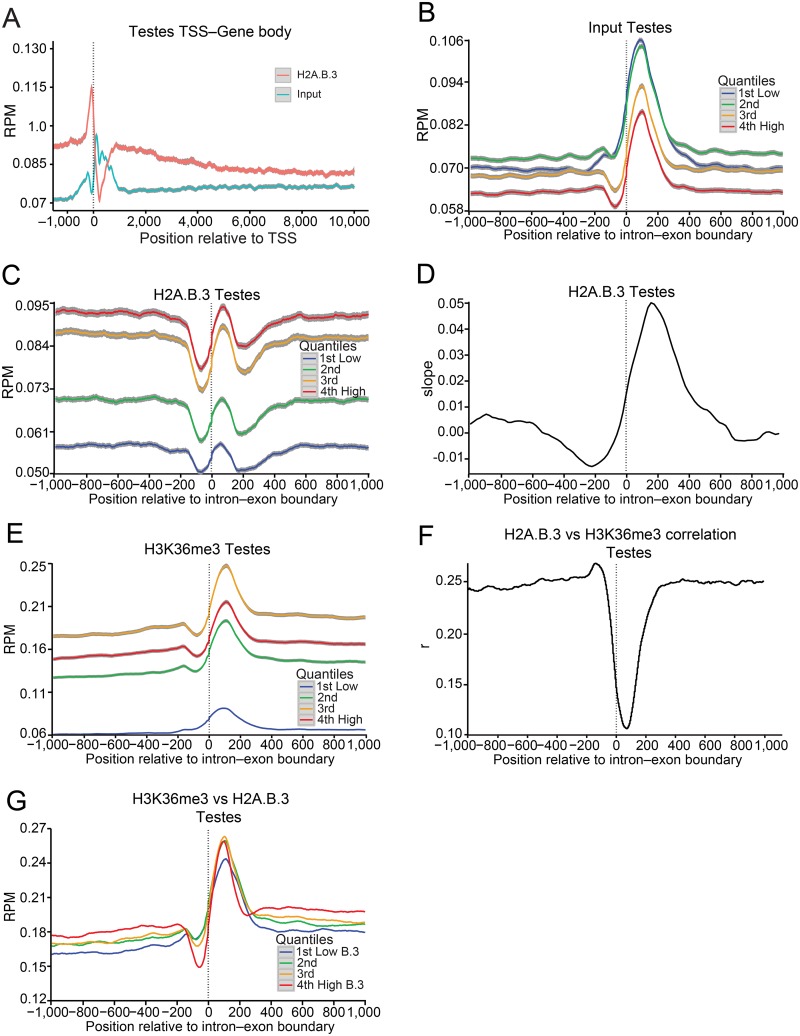
H2A.B.3 is located at the intron—exon boundary of active genes in the testis. Input nucleosomes, nucleosomes immunoprecipitated with H2A.B.3 or H3K36me3 affinity purified antibodies, and poly (A)-transcripts obtained from 28–30 day old mice testes were sequenced yielding 100 base pair paired-end reads (see Material and methods). (a) The individual lines represent the normalised H2A.B.3 and total input reads aligned between -1 and +10 kb from the TSS in the testis. (b) The individual line represents the normalized input nucleosome reads (mean reads per base pair per million reads mapped (RPM)) aligned between -1 and +1 kb from the intron—exon boundary for all exons in the testis ranked according their expression level (repressed, low, medium and high). The colour-map panel shows the relationship between colour and the gene expression rank. (c) Normalized testis H2A.B.3 ChIP-Seq reads ranked according to their expression level aligned with the intron—exon boundary. (d) At each base position relative to the intron exon-boundary, a linear model was fitted to the mean H2A.B.3/input ratio versus gene log expression across all intron—exon boundaries, and the slope of the fitted model plotted for the testis. (e) Normalized testis H3K36me3 ChIP-Seq reads ranked according to their expression level aligned with the intron—exon boundary. (f) Pearson correlation of the log coverage, across 50 base pair windows, was calculated between testes H2A.B.3 ChIP-Seq reads and H3K36me3 ChIP-Seq reads for each base pair relative to the intron—exon boundary. (g) Normalized testis H3K36me3 ChIP-Seq reads ranked according to the incorporation of H2A.B.3 (very low, low, medium and high) aligned with the intron—exon boundary. 95% confidence bands are shown in grey.

First, we produced a testis total input for all genes and a H2A.B.3 ChIP-Seq profile where the normalised reads (mean reads per base pair per million reads mapped (RPM)) at each base pair were aligned with the TSS 1 kb upstream and 10 kb downstream (Fig 1a). It is important to note that H2A.B.3 is not found on all active genes but only a subset ([19]; see below). Several new observations are revealed: (1) H2A.B.3 is located within the gene body but interestingly, H2A.B.3 shows an elevated abundance at the beginning of the gene body compared to the end. This is in contrast to the active gene body mark, H3K36me3, which is more enriched at the end of a gene [20]. (2) On average, H2A.B.3 is ~1.4 times more enriched at the TSS compared to exons. Using a 50 base pair region for the TSS (position -75 to -25 upstream from the TSS) and the intron—exon boundary (from the boundary to 50 base pairs within the exon), the mean coverage in counts per base pair were 19.03 and 13.27, respectively (P-value = 0.03). (3) A marked H2A.B.3 depleted region is observed ~ 225 base pairs downstream of the TSS at the location of the +1 nucleosome.

Next, we investigated whether H2A.B.3 was more enriched on exons or introns, or present on both types of sequences. First, input nucleosomes from mice testes were mapped to intron—exon boundaries (±1 kb) of protein-coding genes and ranked according to their expression level (repressed, low, medium and high). Several studies have shown that exons have an increased nucleosome occupancy compared to introns, thus marking them [21,22]. Consistent with these previous studies, the normalized nucleosome occupancy profile for all exons showed a nucleosome that is strongly positioned at the exon (in mice, most exons are between 50 and 200 base pairs long [23]) (Fig 1b). A negative correlation was observed between the nucleosome occupancy of an exon and the level of expression indicating an overall loss of this nucleosome during transcription (Fig 1b).

Normalized H2A.B.3 ChIP-Seq testis reads were aligned with the intron—exon boundary and indeed an H2A.B.3-containing nucleosome occupying the exon was observed, which was positively correlated with transcription in contrast to the input nucleosome profile (Fig 1c). A plot showing the distribution of input nucleosomes and H2A.B.3 at the intron—exon boundary as a heat map illustrates these transcriptional changes in more detail (S1a and S1b Fig). The H2A.B.3 ChIP-Seq intron—exon plots (Fig 1c) reveals that H2A.B.3-containing nucleosomes are not only located on exons but also on surrounding intronic DNA sequences. Further, a comparison of meta-intron with meta-exon plots shows that H2A.B.3 is distributed throughout the entire intron, and that exons are not enriched with this histone variant compared to intronic sequences (S2a and S2b Fig, Fig 1c). We conclude that in highly expressed genes, H2A.B.3 is enriched in both exon and intron regions and that this enrichment is inversely correlated with nucleosome occupancy seen in the input of the same regions.

While H2A.B.3 was found on both introns and exons, we wondered whether upon transcriptional activation if both exons and flanking intronic sequences gained H2A.B.3 equally well or whether compared to the repressed state, there was a preferential targeting of H2A.B.3 to exons compared to introns. To examine this, we determined the relationship between the H2A.B.3/input ratio and log expression averaged across all intron-exon boundaries. At each base position relative to the intron-exon boundary, a linear model was fit to this relationship and the slope of the fitted linear model was determined and plotted (Fig 1d). The results clearly show that exons gain H2A.B.3 at a higher rate compared to introns when genes are activated.

The exonic H2A.B.3 nucleosome is flanked on both sides by an H2A.B.3 nucleosome depleted region, which is more pronounced at the intron to exon boundary compared to the exon to intron border (Fig 1c). This can also be seen in the H2A.B.3 meta-intron plot (S2a Fig) and when H2A.B.3 ChIP-Seq reads were aligned with the exon—intron boundary (S2c Fig). The H2A.B.3 meta-exon plot revealed that this H2A.B.3 nucleosome is located closer to the exon-intron boundary than the intron-exon boundary (S2b Fig), and accordingly this asymmetry in the H2A.B.3 nucleosome position can provide a simple explanation as to why the intron-exon boundary is more accessible. It is attractive to suggest that this nucleosome-depleted region at the intron—exon boundary could facilitate the access of the spliceosome to the nascent RNA.

The gene body-associated H3K36me3 modification has been shown to be a modifier of splicing outcome [22,24]. To investigate the relationship between H3K36me3, H2A.B.3 and transcription, H3K36me3 ChIP-Seq experiments were performed. As expected, this modification is located at exons and is positively correlated with transcription (although the top 25% of expressed genes display less H3K36me3 compared to moderately expressed genes, Fig 1e). A H3K36me3 heat map further illustrates this positive correlation with expression (S1c Fig).

Next, a Pearson correlation of the log coverage, calculated across 50 base pair windows, was used to determine if there is any correlation between the presence of H2A.B.3 and H3K36me3 at each base pair position relative to the intron-exon boundary (Fig 1f). Intriguingly, no correlation between H2A.B.3 and H3K36me3 at the exon is observed. To examine this relationship further, we separated all intron-exon boundaries into 4 groups that contain very low, low, moderate or high levels of H2A.B.3. For each of the four groups, a single line represents the normalised H3K36me3 reads at each base pair aligned with the intron-exon boundary (Fig 1g). This analysis shows that there is no correlation between the degree of trimethylation at H3K36 with increasing levels of H2A.B.3 incorporation at the exon in the testis. Taken together, with the knowledge that H2A.B.3 and H3K36me3 are enriched at different regions of the gene body, we suggest that H2A.B.3 functions independently from H3K36me3 in the process of gene expression.

In conclusion, based on the observation that H2A.B.3 is found both at the TSS [3] and gene body, including the intron—exon boundary, of an active gene, we suggest that this variant may have more than one role in the process of expressing a gene. Further, given that splicing occurs co-transcriptionally, we explore below the possibility that H2A.B.3 has a role in splicing by determining: (1) whether this histone variant is also found at intron—exon boundaries in the brain, (2) its relationship with the gene body repressive mark H2A.Z, (3) its link with exon inclusion, (4) whether H2A.B.3 interacts with RNA processing factors, (5) whether it can directly interact with RNA and (6) the nuclear localisation of H2A.B.3 and its position in relation to the RNA splicing machinery located at splicing speckles.

### H2A.B.3 is located at the TSS and gene body of actives genes in the brain

H2A.B.3 is also expressed in the mouse brain (S3 Fig) and therefore we wondered whether H2A.B.3 is present at the TSS and gene body of genes active in this tissue. To investigate this, H2A.B.3 ChIP-Seq and RNA-Seq experiments were repeated utilizing the hippocampus and then compared with the testis.

Genes transcribed by RNA polymerase II were separated into groups according to their expression level (repressed, low, medium and high). For each group of genes, a single line represents the normalised tag counts at each base pair, which has been aligned with the start site of transcription (TSS) (±1 kb). Similar to the mouse testis (S4a Fig), a H2A.B.3-containing nucleosome appears at ~ -50 base pairs relative to the TSS with increasing levels of transcription (S4b Fig). Conversely, highlighting the fragile nature of this nucleosome as reported previously [3], and that H2A.B.3 is only present on a subset of active promoters (see below), no input nucleosome is observed at an active TSS both in the testis (S4c Fig) and the hippocampus (S4d Fig). Intriguingly though, the overall H2A.B.3 organisation at an active promoter is different between the testis and the brain. In contrast to the testis, a second H2A.B.3 nucleosome forms at ~ -200 base pairs relative to the TSS on an active promoter (S4b Fig). A plot showing the distribution of H2A.B.3 nucleosomes at the promoter as a heat map for both the testis and hippocampus, respectively illustrates this difference in more detail (S4e and S4f Fig).

Previously, we revealed that H2A.B3 was not present on all active promoters and most interestingly, gene ontology (GO) analyses revealed that H2A.B.3 was particularly enriched on active genes involved in RNA processing and splicing [19]. To investigate this further, gene set enrichment analyses were performed ranked by the mean coverage of H2A.B.3 over a fixed window size of 50 base pairs at successive distances from the TSS (±1 kb). Strikingly, this analysis revealed that the enrichment of H2A.B.3 at the TSS for genes active in the hippocampus displays similar GO terms as active promoters associated with H2A.B.3 in the testis (RNA processing (GO:0006396), translation (GO:0006412) and ribonucleoprotein complex (GO:0030529); S4g and S4h Fig). Importantly, no such functional enrichment at the TSS was observed for input nucleosomes (S4g and S4h Fig). The RNA-Seq data reveals that these H2A.B.3 enriched gene sets show high expression, with a mean RNA-Seq expression 8.2 and 20.7 times greater than the overall mean expression across all genes, for the brain and testes, respectively.

We conclude that H2A.B.3 is a target of the TSS being positioned there both in the testis and the brain. Remarkably, H2A.B.3 is associated with biological functions that are similar in the brain and testis.

H2A.B.3 is also found in the body of active genes in the hippocampus. Recapitulating the observations of the testis, we find that: (1) H2A.B.3 is more enriched at the beginning of the gene body then the end (S5a Fig), (2) the input nucleosome located at the exon is negatively correlated with transcription whereas H2A.B.3 is positively regulated (S5b and S5c Fig), (3) exons gain H2A.B.3 at a faster rate compared to introns when genes are expressed (S5d Fig), (4) incorporation of H3K36me3 at the intron—exon boundary is positively correlated with transcription (S5e Fig) and (5) there is no correlation between the presence of H2A.B.3 and incorporation of H3K36me3 at the intron-exon boundary (S5f and S5g Fig)

Finally, we investigated whether genes that have H2A.B.3 enriched at the TSS also have this histone variant at the intron—exon boundary. To test for this correlated role, we separated all intron-exon boundaries into 4 groups that contain very low, low, moderate, or high levels of H2A.B.3 at the TSS. For each of the four groups, a single line represents the normalised H2A.B.3 reads at each base pair aligned with the intron-exon boundary (S6 Fig). This analysis shows that there is positive correlation between the degree of incorporation at the TSS and the presence of H2A.B.3 at the intron—exon boundary both in the testis and the brain (S6a and S6b Fig). Therefore, transcriptional activation is associated with simultaneous H2A.B.3 incorporation at the TSS and in the intron—exon boundary. This suggests that H2A.B.3 may provide a link between transcriptional initiation and pre-mRNA splicing (see below).

### Histone variant swapping at the intron-exon boundary

H2A.Z is an essential histone variant that is believed to play an important role in establishing an active chromatin structure at promoters [25–28]. However, studies in in plants and *C*.*elegans* have shown that it is also located in the body of genes potentially being involved with repressing gene expression rather than activation [25,29]. To investigate whether H2A.Z is present within the body of genes in the mouse testis and brain, and its link with expression, ChIP-Seq experiments were performed and normalized H2A.Z ChIP-Seq reads were aligned with the intron—exon boundary, which were then ranked according to the level of gene expression.

**Fig 2:**
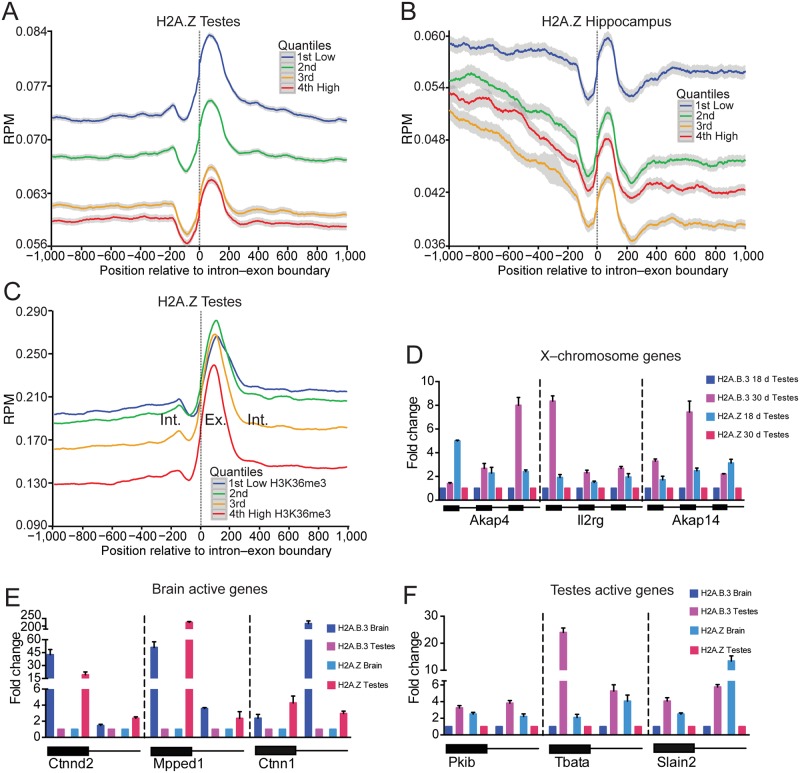
H2A.B.3 replaces H2A.Z on the coding region of active genes. (a) Normalized testis H2A.Z ChIP-Seq reads ranked according to their expression level (repressed, low, medium and high) aligned with the intron—exon boundary. (b) Normalized hippocampus H2A.Z ChIP-Seq reads ranked according to their expression level aligned with the intron—exon boundary. (c) Normalised testis H2A.Z ChIP-Seq reads ranked according to the incorporation of histone H3 trimethyl K36 (very low, low, medium and high) aligned with the intron—exon boundary. (d) Quantitative H2A.B.3 and H2A.Z ChIP assays were performed in the testis at three exons for each of three individual genes (*Akap4*, *Il2rg* and *Akap14*) located on the X chromosome that are repressed at day 18 (the pachytene stage) but activated at day 30 (the late round spermatid stage). Standard deviation of three replicates is shown. (e) Quantitative H2A.B.3 and H2A.Z ChIP assays were performed in the hippocampus at an exon and its neighbouring intronic sequences for genes that are expressed more highly in the brain (*Ctnnd2*, *Mpped1* and *Ctnn1*) versus the testis (a brain to testis expression ratio of 27.1, 17.4 and 75.4, respectively). Standard deviation of three replicates is shown. (f) Quantitative H2A.B.3 and H2A.Z ChIP assays were performed in the testis at an exon and its neighbouring intronic sequences for genes that are expressed more highly in the testis (*Pkib*, *Tbata* and *Slain2*) versus the brain (a testis to brain expression ratio of 7.3, 23.4 and 22.2, respectively). Standard deviation of three replicates is shown.

Significantly, a H2A.Z-nucleosome was observed on the exon and in contrast to H2A.B.3, H2A.Z was negatively correlated with transcription (Fig 2a and 2b). A histone H2A.Z heat map illustrates this negative correlation with expression clearly (S1d Fig). Not surprisingly then, there was also a negative correlation between H2A.Z incorporation at the intron—exon boundary with increasing levels of H3K36me3 (Fig 2c). This result raises the intriguing possibility that there is a dynamic histone variant replacement process whereby during the activation of transcription, H2A.Z is lost from the intron-exon boundary being subsequently replaced with H2A.B.3.

To demonstrate directly that H2A.B.3 can replace H2A.Z on the same gene when it becomes activated during development, we used published gene expression data from Namekawa and colleagues [30] where they identified a small number of developmentally regulated genes on the X chromosome. These genes are repressed at the pachytene stage (day 18) but become activated in round spermatids (day 30). We chose three such genes (*Akap4*, *Il2rg* and Akap14) and performed quantitative H2A.B.3 and H2A.Z ChIP assays to examine the relative amount of these histone variants at 3 different exons for each gene (Fig 2d). For all exons in all genes, the level of H2A.Z decreases with a corresponding increase in H2A.B.3 when the genes become activated in round spermatids. Using these representative examples, these results clearly demonstrate that H2A.Z is associated with repressed genes in the coding region and upon transcriptional activation, H2A.B.3 replaces it (noting that we previously demonstrated that the targeting of H2A.B.3 to these X-linked genes was concurrent with gene activation [3]).

Next, quantitative H2A.B.3 and H2A.Z ChIP assays were performed examining the relative amount of these histone variants at exons and neighbouring intronic sequences of genes either expressed more highly in the brain (*Ctnnd2*, *Mpped1* and *Ctnn1*) or in the testis (*Pkib*, *Tbata* and *Slain2*) (Fig 2e and 2f). In all cases, the highest level of H2A.B.3 at intron—exon sequences occurred when the gene was active irrespective of whether it is expressed in the brain or the testis (Fig 2e and 2f). Conversely, all genes contain more H2A.Z when they were not expressed. As examples, *Mpped1* has ~ 50 fold more H2A.B.3 at its exon in the brain (where it is expressed) compared to the testis, (Fig 2e). On the other hand, this exon has ~ 250 times more H2A.Z in the testis where this gene is not expressed compared to the brain (Fig 2f). Similarly, *Tbata* has ~ 25 fold more H2A.B.3 at its exon in the testis where it is expressed (Fig 2f) while it has 2 fold more H2A.Z in the brain where it is not expressed (Fig 2e). These findings show that in a tissue specific manner, H2A.B.3 replaces H2A.Z when a gene becomes activated verifying the genome-wide observations (Fig 1c, S5c Fig, Fig 2a and 2b).

### A positive correlation between the incorporation of H2A.B.3 and exon inclusion

**Fig 3:**
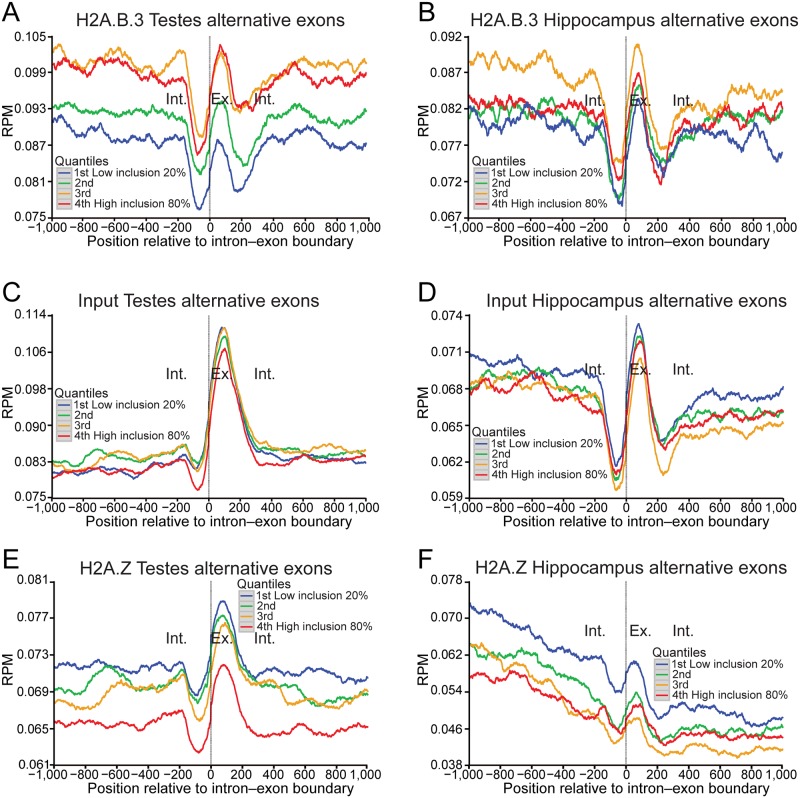
The incorporation of H2A.B.3 at alternatively spliced exons is positively correlated with the level of inclusion. (a) Normalised testis H2A.B.3 ChIP-Seq reads aligned with the intron—exon boundary ranked according to the level of inclusion of alternatively spliced exons (very low, 20%; low, 40%; medium, 60%; and high, 80%). (b) Normalised hippocampus H2A.B.3 ChIP-Seq reads aligned with the intron—exon boundary ranked according to the level of inclusion of alternatively spliced exons. (c) Normalised testis input reads aligned with the intron—exon boundary ranked according to the level of inclusion of alternatively spliced exons. (d) Normalised hippocampus input reads aligned with the intron—exon boundary ranked according to the level of inclusion of alternatively spliced exons. (e) Normalised testis H2A.Z ChIP-Seq reads aligned with the intron—exon boundary ranked according to the level of inclusion of alternatively spliced exons. (f) Normalised hippocampus H2A.Z ChIP-Seq reads aligned with the intron—exon boundary ranked according to the level of inclusion of alternatively spliced exons.

The brain, followed by the testis, displays the greatest level of alternative splicing compared to any other tissue [31]. The next obvious question to address was whether the gain of H2A.B.3 at the intron—exon boundary has a potential role in splicing or whether it is only linked to the process of transcriptional elongation as suggested by previous *in vitro* experiments [4]. To distinguish between these possibilities, we ranked all alternatively spliced exons into 4 groups dependent upon their inclusion levels (very low, low, moderate or high; noting that alternatively spliced exons represent a minor population (13.4%) compared to constitutive included exons) (Fig 3). Significantly, a clear positive correlation exists between the degree of exon inclusion and the level of H2A.B.3 at the intron—exon boundary in the testis with a similar trend in the hippocampus (Fig 3a and 3b) whereas input nucleosomes do not (Fig 3c and 3d). Conversely, H2A.Z nucleosomes are negatively correlated with exon inclusion (Fig 3e and 3f). H2A.B.3 is not only found on alternatively spliced exons but is also present on constitutively included exons (S7 Fig). These data argue that the presence of H2A.B.3 at the intron—exon boundary has a role in the pre-mRNA splicing process.

#### H2A.B.3 interacts with Pol II and RNA processing and export factors

If H2A.B.3 does have a role in splicing, then it would be predicted that this histone variant would interact with splicing factors. An additional prediction would also be that H2A.B.3 would associate with the initiation form of RNA Pol II (SerPhos5) given its location at the TSS.

To identify H2A.B.3 nucleosome interacting proteins, an unbiased proteomics approach was used. This involved isolating H2A.B.3-containing nucleosomes at the time when H2A.B.3 is maximally expressed (from the testes of mice 28–30 days old) following the same procedure as used for ChIP-Seq experiments. Co-purified proteins were then analysed by LC-MS/MS following stringent washing of immunoprecipitated H2A.B.3 nucleosomes (1M NaCl in wash buffer, see Methods for details). As a rigorous control to remove protein contaminants, mass spectrometry analysis was also performed on H2A.Z-containing nucleosomes. Using this strategy, the genomic (and nuclear, see below) locations of H2A.B.3 can be linked with the function(s) of putative proteins that specifically interact with H2A.B.3 nucleosomes.

Following two independent H2A.B.3 or H2A.Z immunoprecipitations (IPs) and MS analyses, the cumulative data was first filtered for common protein contaminants using the www.crapome.org data base, followed by removal of all proteins common between H2A.Z and H2A.B.3. Next, only those proteins present in both IP H2A.B.3 replicates were used to generate a list of proteins that preferentially interact with H2A.B.3 nucleosomes. We identified 33 proteins that co-purified with H2A.B.3 and not H2A.Z and the majority of these (25/33) were involved in pre-mRNA processing, transcription and most interestingly, the piRNA pathway (which will be the subject of future investigations) (Table 1 and S1 Table). As expected, subunits of the RNA Pol II complex were identified. Significantly, Spt6 was also immunoprecipitated with H2A.B.3. Spt6 enhances transcriptional elongation by RNA Pol II by functioning as a histone H3–H4 chaperone. It also has an important role in ensuring proper mRNA splicing and export [32]. The histone proteins that co-immunoprecipitated with H2A.B.3 forming this nucleosome are also listed (which, in this case, showed no differences compared with the histones that immunoprecipitated with H2A.Z, S2 Table).

**Table 1:** RNA-binding proteins that co-immunoprecipitate with anti-H2A.B.3 but not with ant- H2A.Z antibodies.

ACCESSION ID	PROTEIN NAME	GENE NAME	# UNIQUE PEPTIDES	MW (KDA)	RNA-RELATED PROCESS
**3’-UTR processing**					
P60824	Cold-inducible RNA-binding protein	Cirbp	5	18.6	3'-UTR-binding
Q99LI7	Cleavage stimulation factor subunit 3	Cstf3	2	82.8	Polyadenylation/3'-end cleavage
Q64368	Deleted in azoospermia-like*	Dazl	1	33.3	3'-UTR binding
P16858	Glyceraldehyde-3-phosphate dehydrogenase	Gapdh	2	35.8	3'-UTR binding
Q60668	Heterogeneous nuclear ribonucleoprotein D0	Hnrnpd	3	38.3	3'-UTR binding
Q80X82	Symplekin	Sympk	3	142.2	Scaffold subunit of the 3'-UTR-processing complex
P52912	Nucleolysin TIA-1*	Tia1	1	42.8	3'-UTR binding/alternative splicing
**Splicing**					
Q62376	U1 small nuclear ribonucleoprotein 70 kDa	Snrnp70	7	52	Spliceosome subunits
P57784	U2 small nuclear ribonucleoprotein A'	Snrpa1	4	28.3	Spliceosome subunits
P62309	Small nuclear ribonucleoprotein G	Snrpg	2	8.5	Spliceosome subunits
Q6PE01	U5 small nuclear ribonucleoprotein 40 kDa	Snrnp40	5	39.3	Spliceosome subunits
Q62189	U1 small nuclear ribonucleoprotein A U1A	Snrpa	4	31.8	Spliceosome subunits
Q810A7	ATP-dependent RNA helicase DDX42	Ddx42	2	101.9	Splicing factor
Q4FK66	Pre-mRNA-splicing factor 38A*	Prpf38a	1	37.4	Splicing factor
Q6NV83	U2 snRNP-associated SURP containing protein	U2surp	6	118.2	Splicing factor
G5E866	Splicing factor 3B subunit 1 SF3b155	Sf3b1	5	145.7	Splicing factor
Q8VDW0	ATP-dependent RNA helicase DDX39A	Ddx39a	4	49	pre-mRNA splicing/RNA export
**Exon Junction/Nonsense Mediated Decay**					
Q9EPU0	Regulator of nonsense transcripts 1#	Rent1	1	123.9	RNA-dependent helicase/ATPase required for NMD
O55128	Histone deacetylase complex subunit Sap18	Sap18	4	17.6	Auxiliary component of EJC/transcriptional repression
**Transcription**					
Q62383	Transcription elongation factor SPT6	Spt6	3	199	Transcription enhancer/histone chaperone/RNA splicing
P08775	DNA-directed RNA polymerase II subunit RPB1	Polr2a	5	217	Transcription/RNA splicing
Q8CFI7	DNA-directed RNA polymerase II subunit RPB2	Polr2b	3	133.8	Transcription/RNA splicing
**piRNA pathway**					
Q99MV1	Tudor domain-containing protein 1	Tdrd1	2	129.6	piRNA proccessing
P61407	Tudor domain-containing protein 6	Tdrd6	2	237.8	piRNA proccessing
Q9JMB7	Piwi-like protein 1	Piwil1	8	98.5	piRNA proccessing

Mononucleosomes were prepared from the testis of 28–30 day old mice and immunoprecipitated with anti-H2A.B.3 or ant- H2A.Z antibodies. Mass Spectrometry then identified the co-immunoprecipitated proteins. To exclude non-specific protein hits, only proteins that were identified in two H2A.B.3 biological replicas and not present in either of the two H2A.Z biological replicas were considered as a H2A.B.3 interacting protein. 25/33 proteins were involved in RNA processing and transcription as shown.

* These three protein hits are of lower confidence.

^#^ Rent1 was confirmed by Western Blot analysis.

**Fig 4:**
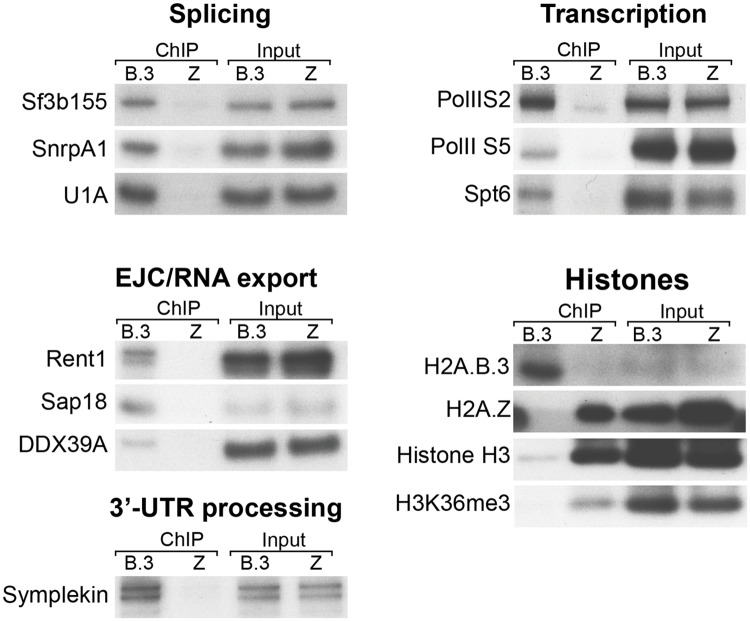
H2A.B.3 co-immunoprecipitates with proteins involved in RNA processing and transcription. Mononucleosome-enriched chromatin from 28–30 day old mouse testes was immunoprecipitated with anti-H2A.B.3 or anti-H2A.Z antibodies. Co-immunoprecipitated proteins were identified by subsequent western blotting with the indicated antibodies selected to detect proteins involved in different aspects of RNA synthesis, processing and export. The presence of H2A.B.3, H2A.Z, histone H3 and H3K36me3 in the H2A.B.3 or H2A.Z co-immunoprecipitate were also examined by western blotting. Input chromatin (1/20 of the amount used for a ChIP-Seq experiment) was used as a loading control.

To validate the interaction between H2A.B.3 nucleosomes and the above factors involved in pre-mRNA processing, post-splicing processes and transcription, IPs of H2A.B.3 and H2A.Z-containing nucleosomes were repeated and a Western blot analysis was performed using commercially available antibodies against key proteins involved in these different processes (Fig 4). Proteins required for splicing, 3’–UTR processing and export as well as proteins present in the Exon Junction complex clearly co-immunoprecipitated with H2A.B.3 nucleosomes but not with H2A.Z nucleosomes (Fig 4). Phosphorylation of the RNA Pol II CTD domain at the serine 5 position occurs upon promoter clearance. Serine 2 phosphorylation is important for mRNA elongation, splicing and 3'-end processing [33]. Significantly, and in contrast to H2A.Z, both phosphorylated forms were immunoprecipitated with H2A.B.3 as was Spt6, consistent with this histone variant being located at the TSS and the body of active genes (Fig 4). In summary, it appears that H2A.B.3 activates the expression (S4g and S4h Fig) of the RNA processing factors that it physically interacts with (Table 1, Fig 4).

An important fact that needs to be emphasized is that H2A.B.3 is considerably less abundant in chromatin compared to H2A.Z (which accounts for the low histone H3 western blot signal in Fig 4). At the late round spermatid stage, H2A.Z expression is ~30-fold higher (S8 Fig). Analysis of the RNA-Seq data reveals that H2A.B.3, H2A.Z, and all of the remaining H2A’s were expressed with a mean TPM (transcripts per million) of 0.65, 16.85 and 1.06, respectively. Further, H2A.B.3 accounts for 3.7% of the total pool of H2A (including H2A.Z). This reinforces the view that the association between H2A.B.3 and pre-mRNA processing factors and RNA Pol II is specific and physiologically relevant (since no interactions between H2A.Z and RNA processing factors are observed even though H2A.Z is more highly expressed). Taken together, these data provides further support that H2A.B.3 has a new pre-mRNA processing role during mouse spermatogenesis.

#### H2A.B.3 directly interacts with RNA in vitro and in vivo

The finding that H2A.B.3 interacts with RNA processing factors and RNA Pol II raises two important questions. First, which interactions are direct and which are not given that chromatin was first crosslinked before the immunoprecipitation of H2A.B.3-containing nucleosomes and subsequent mass spec analysis. In other words, given that all proteins involved in transcription and splicing exist as complexes, some of the proteins identified in Table 1 may be the result of indirect protein interactions captured by crosslinking. Second, given that many RNA processing proteins are ribonucleoproteins, are the interactions between H2A.B.3 and the RNA processing factors (along with RNA Pol II) mediated by protein-protein associations or by RNA-protein interactions?

**Fig 5:**
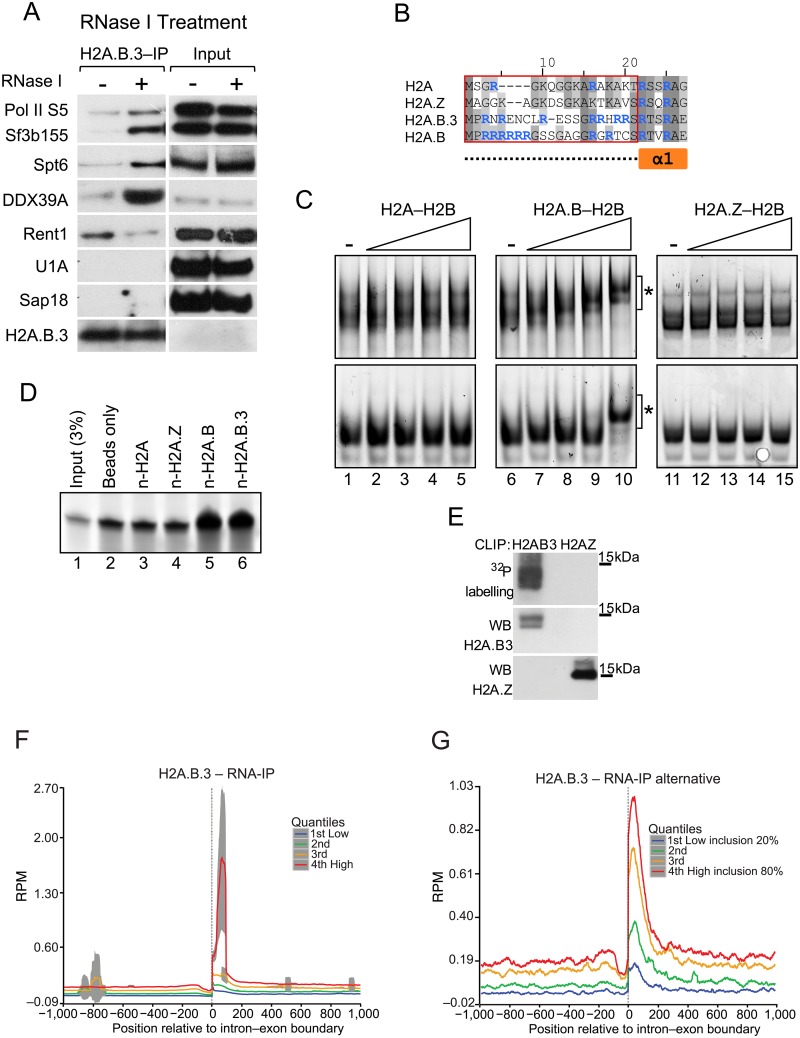
H2A.B.3 can bind RNA *in vitro* and *in vivo*. (a) Total cellular lysates were prepared from UV treated mouse testes and treated with RNase I or not. H2A.B.3 was then immunoprecipitated and the co-immunoprecipitated proteins were identified by western blotting with the indicated antibodies selected to detect proteins involved in different aspects of RNA synthesis, processing, and export. (b) Amino acid sequence alignment of the N-terminal region of histone H2A and the variants H2A.Z, H2A.B.3, and H2A.B. Compared to H2A, the N-terminus of H2A.B.3 and H2A.B are 6.3% and 23.5% identical, respectively. The red box demarcates the sequences corresponding to the N-terminal peptides used for the pulldown experiments in panel d, and corresponds to the unstructured region (dashed line) preceding the first alpha helix of H2A (α1; orange box). Arginine residues are highlighted in blue. (c) Histone dimer samples (0.6, 1.1, 2.3, 4.5 μM) were incubated with 20 ng *in vitro* transcribed RNA (222 nt and 152 nt, top and bottom panels, respectively) and analysed on 5% acrylamide 1X TB gels. The asterisk (*) denotes shifted bands corresponding to H2A.B—H2B-RNA complexes. (d) An RNA pulldown assay using biotinylated histone N-terminal peptides (n-H2A, n-H2A.Z, n-H2A.B and n-H2A.B; 130 pmol). Samples were run on 15% TBE-Urea gels, along with input RNA (5 pmol; 3% of total input) for comparison. (e) CLIP assays demonstrating that H2A.B.3 but not H2A.Z directly interacts with RNA in germ cells. Also show is the western blot analysis of the immunoprecipitated H2A.B.3 and H2A.Z. Following the RNA—IP procedure (see Methods), cells isolated from 28–30 day old testes were UV crosslinked, the chromatin sheared and following the immunopurification of H2A.B.3-containing chromatin fragments, the released RNA was sequenced to yield 100 base pair paired end reads. (f) H2A.B.3 RNA plot ranked according to expression aligned with all intron—exon boundaries. (g) A H2A.B.3 RNA plot ranked according to the level of exon inclusion (20 to 80%) aligned with the intron—exon boundary of alternatively spliced exons.

To address these two questions, we prepared total cellular lysates from 28–30 day old mice testes followed by crosslinking of RNA to proteins with UV light. Following this, half of the lysate was subjected to RNase I digestion while the other half was not. Next, H2A.B.3 was immunoprecipitated and the interaction with selected processing and export factors (Sf3b155, Spt6, DDX39A, Rent1, U1A and Sap18) as well as RNA Pol II were investigated by Western Blot analysis (Fig 5a). No interactions between U1A and Sap18 were observed indicating that their interactions with H2A.B.3 were not direct.

Unexpectedly, interactions between Sf3b155, Spt6, DDX39A and RNA Pol II, and H2A.B.3 were dramatically enhanced following RNase I digestion. In contrast, the interaction with Rent1 was reduced. This suggests that RNA has an important role in modulating the ability of H2A.B.3 to interact with these factors. One attractive hypothesis that can account for these observations is that H2A.B.3 is in fact a RNA binding protein so that RNA can function as a competitive inhibitor for its interaction with Sf3b155, Spt6, DDX39A or RNA Pol II. On the other hand, the interaction between H2A.B.3 and Rent1 is dependent upon RNA.

The N-terminal tails of both H2A.B.3 and H2A.B lack lysine residues but are enriched with arginine residues, and a common feature of RNA binding proteins is the preponderance of arginine residues in their unstructured regions [34]. Further, the greatest divergence of amino acid sequence between H2A and H2A.B/H2A.B.3 occurs in the N-terminal regions (Fig 5b). To investigate whether H2A.B/H2A.B.3 are indeed RNA binding proteins, we performed electrophoretic mobility shift assays using two single stranded diverse RNA probes (222 nt and 152 nt) that were previously used to assess whether specific proteins possess RNA-binding properties [35]. We compared the relative RNA binding ability of the H2A.B—H2B dimer *versus* H2A—H2B and H2A.Z—H2B dimers (unfortunately, under the conditions of the assay, the H2A.B.3–H2B dimer was insoluble). Strikingly, H2A.B—H2B dimers bound to both RNA probes, whereas H2A—H2B and H2A.Z—H2B dimers did not (Fig 5c). Importantly, the affinity of the H2A.B—H2B:RNA interaction is comparable to other known protein:RNA interactions, as the range of concentrations (micromolar) that we observe binding is similar to those previously reported for *bona fide* RNA binding proteins using this assay [35].

To investigate whether indeed the N-terminal tails of H2A.B and H2A.B.3 have acquired the ability to bind RNA directly, we performed RNA-pulldown assays (with the RNA probe in excess, see Methods) using biotinylated N-terminal peptides for H2A, H2A.Z, H2A.B and H2A.B.3 (Fig 5d; the peptide sequences used are denoted by the red box in Fig 5b). RNA was consistently enriched in pulldowns using the H2A.B and H2A.B.3 peptides, whereas neither the H2A nor H2A.Z peptides bound RNA above background levels. We conclude that the N-terminal tails of both H2A.B and H2A.B.3 possess a novel RNA binding ability. This supports our hypothesis that the interaction of H2A.B.3 with RNA could competitively inhibit the ability of H2A.B.3 to interact with RNA processing factors and RNA Pol II.

To investigate whether H2A.B.3 can directly interact with RNA *in vivo*, we performed CLIP assays (UV Cross-Linking of RNA to chromatin and Immuno-Precipitation of histone variant-containing nucleosomes) using cellular lysates from 28–30 day old mice testes as described for Fig 5a. Following the treatment of lysates with RNase I, H2A.B.3 and H2A.Z (as a control) were immunoprecipitated and any remaining RNA that was protected by the interaction with the histone variant was radioactively labeled. Fig 5e clearly shows that H2A.B.3 (which runs as a doublet) but not H2A.Z binds directly with RNA *in vivo*. We conclude that H2A.B.3 is a unique histone variant being able to interact with non-histone proteins (RNA processing factors), DNA as well as RNA.

To investigate whether the RNA that associates with H2A.B.3 in round spermatids maps to the intron—exon boundary, we performed RNA-H2A.B.3 imunoprecipitation (RNA-IP) Seq experiments. RNA was first crosslinked to chromatin with UV light and then H2A.B.3-containing nucleosomes were immunoprecipitated following sonication. The resultant RNA—IP library was then sequenced yielding 100 base pair paired-end reads and mapped to the intron—exon boundary (Fig 5f and 5g). Importantly, as a control to remove non-specific RNA-protein interactions, we also performed RNA-IP on immunopurified H2A.Z nucleosomes (see Materials and Methods).

H2A.B.3 does associate with transcript RNA and this RNA represents the mature transcript because it maps to exonic but not intronic sequences (Fig 5f). Further, this RNA maps closer to the intron—exon boundary (65 base pairs) than to the exon—intron boundary (115 base pairs) when examining the average pattern for all exons. Importantly, this association of H2A.B.3 with RNA is strongly correlated with high levels of gene expression (Fig 5f) and the inclusion of alternatively spliced exons (Fig 5g) as expected if H2A.B.3 does have a role in splicing. On the other hand, H2A.B.3 does not associated with RNA at the TSS (S9 Fig). When overlapping RNA-IP data with H2A.B.3 ChIP-Seq data over gene bodies and promoters (-1000 bp upstream of TSS), the RNA-IP covers 4.6% of H2A.B.3 ChIP-Seq reads (see Materials and Methods).

#### H2A.B.3 is found at splicing speckles

Nuclear or splicing speckles are highly dynamic nuclear compartments necessary for splicing but their link with transcription and splicing appears to be controversial. One view is that they function as a storage and assembly site for pre-mRNA splicing factors, 3’-end RNA processing factors and mRNA export factors and as such, contains no or very little DNA [36]. On the other hand, it has been suggested that active splicing, and perhaps even transcription, are associated with these nuclear bodies [37,38]. Given that H2A.B.3 is a component of chromatin that interacts with RNA processing factors, we examined the relationship between H2A.B.3 and splicing speckles. To investigate this, we performed indirect immunostaining of surface-spread preparations of round spermatids with affinity purified H2A.B.3 antibodies and antibodies against the splicing speckle marker Y12, splicing factors (SnrpA1, Sf3b155 and U1A) and Spt6. DNA was stained with DAPI.

**Fig 6:**
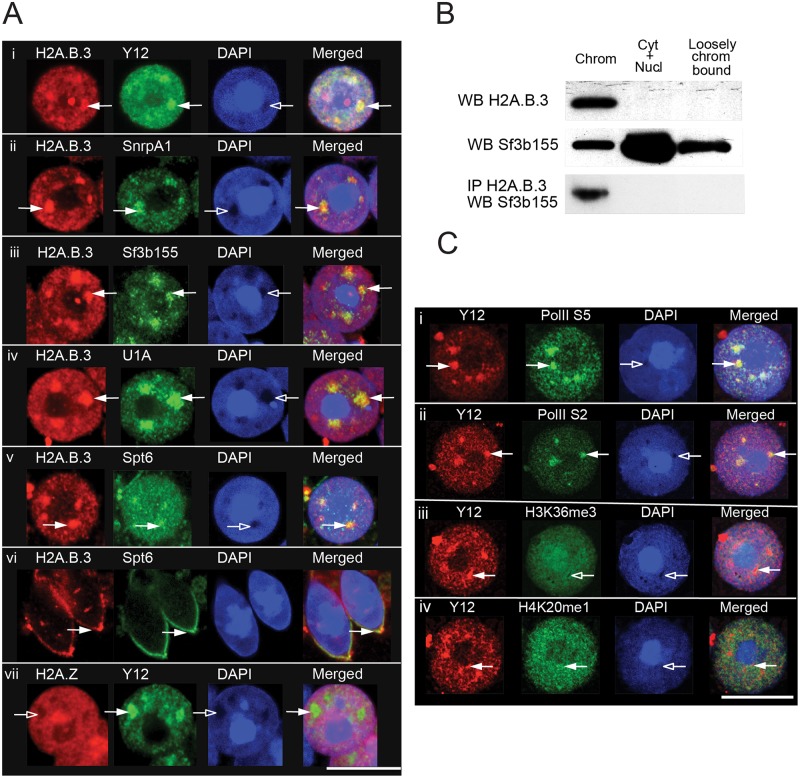
H2A.B.3 co-localises with proteins involved in RNA processing and transcription. (a) Hypotonic spreads of germ cells from adult mouse testis showing round (i-v, vii) and elongating spermatids (vi) were indirectly immunostained with antibodies against H2A.B.3 (i-vi) or H2A.Z (vii), the splicing speckle marker, Y12 (i and vii) and representative RNA-binding proteins that co-immunoprecipitated with H2A.B.3 in panel a (ii-vi). White arrows show splicing speckles. Scale bar, 10μm. (b) Round spermatids were isolated by gravity sedimentation and then fractionated into a chromatin fraction (chrom), a cytoplasmic and nucleoplasmic fraction (Cyt+Nucl) and a loosely bound chromatin faction (loosely chrom bound). (c) Hypotonic spreads of round spermatids from adult mouse testis were stained with DAPI and indirectly immunostained with antibodies against the splicing speckle marker, Y12 (i-iv), initiating and elongating states of RNA Pol II S5 (i), and S2 (ii) respectively, H3K36me3 (iii), H4K20me1 (iv). White arrowheads show accumulation of a signal in splicing speckles, empty arrowheads show depletion. Scale bar, 10μm.

Strikingly, in all round spermatids that were examined (> 1000), large H2A.B.3 foci exist that co-localise with Y12, SnrpA1, Sf3b155, U1A and Spt6 (Fig 6a) showing that H2A.B.3 is indeed found at splicing speckles. Further, to our knowledge, the presence of Spt6 at splicing speckles has not been previously reported. Both Spt6 and H2A.B.3 were exported together out of the nucleus when round spermatids differentiate into elongating spermatids, which is when transcription is shut down (Fig 6a, panel vi). As expected H2A.Z was not located at splicing speckles (Fig 6a, panel vii). Another interesting feature of these enriched domains of H2A.B.3 is that they are located at regions of DAPI (DNA) depletion (Fig 6a, see arrows).

To investigate whether these H2A.B.3 enriched—DNA depleted regions represent highly decompacted domains of chromatin or signify a non-chromatin bound form of H2A.B.3, we performed biochemical cell-fractionation experiments. Round spermatids were first isolated by BSA gradient sedimentation and then subcellular fractionated into a cytoplasmic and nucleoplasmic fraction, a loosely bound chromatin fraction and a tightly bound chromatin fraction (Fig 6b). Western Blot analysis revealed that the bulk of H2A.B.3 is tightly associated with chromatin with no H2A.B.3 present in the cytoplasmic and nucleoplasmic fractions (although it is possible that a small pool of chromatin unbound H2A.B.3 exists, which is not detected by this western analysis). This observation that the bulk of H2A.B.3 is chromatin bound argues that the H2A.B.3 enriched—DNA depleted regions seen in round spermatids are indeed highly decompacted domains of chromatin, which is consistent with its ability to decondense chromatin *in vitro* [3,4].

The splicing factor Sf3b155, on the other hand, is found in all three fractions but being most enriched in the cytoplasmic and nucleoplasmic fraction (Fig 6b). Confirming that it is chromatin bound H2A.B.3 that interacts with splicing factors, immunoprecipitation of Sf3b155 using affinity purified H2A.B.3 antibodies only occurred in the tightly bound chromatin fraction (Fig 6b). Taken together, these observations suggest that splicing speckles form at highly decompacted domains of chromatin that contain H2A.B.3. Further, both active forms of RNA Polymerase II (RNA Pol II—SerPhos5 and—SerPhos2) are located at these splicing speckles supporting the view that indeed these nuclear domains can be located at transcribed regions of the genome (Fig 6c). This is confirmed by the location of the active modifications H4K20me1 (an active promoter mark) and H3K36me3 within or at the periphery of splicing speckles. We therefore conclude that highly transcriptional active round spermatids contain novel H2A.B.3–enriched nuclear domains that are transcriptional active, which co-localize within splicing speckles.

## Discussion

No study to date has examined the function of H2A.B.3 in its proper physiological contexts i.e. both in the testis and brain. Here we addressed this issue and have uncovered new locations for this histone variant on an active gene. The observed enrichment of H2A.B.3 at the TSS and the beginning of the gene body is distinctively different compared to any other type of chromatin modification. Evidence is provided that H2A.B.3 not only has a function in the initiation of transcription in the testis [3] (Table 1, Fig 4) and the brain (S4 Fig), but also has a role in the processing of RNA (Figs 3, 4 and 5f, Table 1) thus providing a new link between transcriptional initiation and splicing. An unexpected histone variant replacement process was also uncovered whereby H2A.B.3 replaces H2A.Z at intron—exon boundaries when a gene becomes active (Fig 2d–2f). Previously, we reported that H2A.B.3 might also replace H2A.Z at the TSS leading to higher levels of transcription suggesting that this histone variant replacement process may not be limited to intron—exon boundaries [19]. This is also consistent with the observation that *in vitro*, H2A.Z-containing nucleosome arrays are more refractory to transcription then H2A-containing arrays[4]. To date, H2A.Z has largely been viewed as an activator of transcription with its main function to assemble the TSS into an active chromatin structure [17,25–28]. Our results suggest that H2A.Z may have a different repressive function when incorporated into the body of a gene in the testis and brain.

We also reveal a novel nuclear organisation in round spermatids where distinct and large domains of highly decondensed H2A.B.3-containing chromatin exist, which co-localise with splicing speckles. Further, these H2A.B.3-containing domains appear to be transcriptionally active based on its co-localisation with the initiation and elongation forms of RNA Pol II (Fig 6). This suggests that splicing speckles are not simply passive sites for the storage of splicing factors but participate in the transcription process, at least in highly transcriptionally active round spermatids. The presence of H2A.B.3 at highly decondensed domains of chromatin in round spermatids is consistent with the *in vitro* ability of H2A.B.3 to destabilise the nucleosome and inhibit chromatin compaction [3,4].

Perhaps the most unexpected finding of this study is the observation that H2A.B/H2A.B.3 are RNA binding proteins consistent with a role in RNA processing and its association with mature transcripts *in vivo* (Fig 5). Further, this RNA binding ability appears to negatively regulate its capacity to interact with RNA Pol II and certain other RNA processing factors suggesting that a competition may exist between its capacity to bind to proteins or RNA. On the other hand, the interaction of at least one factor, Rent, was dependent upon RNA. While no representative RNA binding module exists, a common feature is a preponderance of arginine residues, which commonly occurs with serine and/or glycine residues [34]. These features are observed in the N-terminal tails of H2A.B/H2A.B.3 (Fig 5b). We conclude that H2A.B.3 is a unique histone variant being able to bind to both RNA and DNA. It is attractive to speculate that these roles of H2A.B.3 enables a special transcription/splicing mechanism to operate in the testis and the brain, two tissues known to display the highest level of splicing compared to other cell types [31] (see below).

What makes H2A.B.3 a truly remarkably histone though, is not only its ability to bind to RNA but also its capacity to directly interact with proteins. Our mass spec analysis of immunoprecipitated H2A.B.3-containing nucleosomes revealed an interaction with proteins involved in transcription and RNA processing. However, it was unclear whether these H2A.B.3-protein interactions were direct because the chromatin was first cross-linked with formaldehyde. Therefore, we repeated these experiments without formaldehyde crosslinking followed by the mechanical shearing of germ cell nuclei (Fig 5a). We demonstrated that indeed H2A.B.3 could directly interact with RNA Pol II, Spt6 and other splicing factors (which was greatly enhanced by the removal of RNA). On the other hand, the interactions with U1A and Sap18 were lost indicating that these interactions were indirect.

A recent study over expressed epitope-tagged H2A.B in HeLa cells [15]. Perhaps not surprisingly, significant differences in H2A.B chromatin organisation are observed between Hela cells and what is observed here in the testis and brain (major differences are seen at the TSS, at the beginning of the gene body and at the intron-exon boundary, and no role for RNA in regulating H2A.B-splicing factor interactions was observed). While also not observing interactions with RNA Pol II, Spt6 and many other factors, Tolstorukov and colleagues did observe an interaction between H2A.B and certain splicing factors (but noting that, as shown here, some of these interactions may not be direct). Now in combination with our observations, this suggests that H2A.B has the intrinsic ability to interact with splicing factors even when expressed in a non-physiological setting, which could have important implications for the understanding of certain types of cancers [16].

A more recent study suggested that H2A.B.3 is specifically deposited to methylated CpGs within the gene body in mouse ES cells to overcome methylation mediated repression of transcriptional elongation. While we cannot rule out that H2A.B.3 may have a similar role in the testis and the brain, our results suggest that H2A.B.3 may have a more universal role in facilitating gene expression that includes non-methylated DNA regions given our finding that H2A.B.3 replaces H2A.Z during the gene activation process. At a genome-wide level, it has been clearly established that H2A.Z and DNA methylation are mutually antagonistic chromatin marks [39]. Further, for reasons that are unclear, this study also did not observe H2A.B.3 at the TSS but this does suggest that mouse ES cells (and Hela cells) lack the H2A.B.3 targeting mechanisms that operate in the testis and brain.

As noted above, previously we demonstrated that H2A.B/H2A.B.3 could destabilise the nucleosome, inhibit chromatin compaction and thus promote transcription *in vitro* [3,4]. Given that H2A.B.3 is located at the TSS as well as in the body of an active gene in the testis and the brain, we suggest that these biophysical properties of H2A.B chromatin facilitates both transcription initiation and elongation. During spermatogenesis, the highest overall level of gene expression occurs at the round spermatid stage [18], which correlates when H2A.B.3 is maximally expressed. Previously, we showed that the targeting of H2A.B.3 to the TSS, in a stage specific manner, is concurrent with the activation of previously silent genes on the X chromosome, and genes that were previously active became more highly expressed in round spermatids [3,17,19]. Here, we show that the exons of previously inactive X chromosome genes also gain H2A.B.3 when they become activated in round spermatids, which is consistent with the observation that H2A.B.3 incorporation at the TSS occurs concurrently with its deposition at the intron—exon boundary (S6 Fig). On average, the abundance of H2A.B.3 is the highest at the TSS compared to the gene body, and intriguingly, within the gene body, its occupancy is greatest at the beginning of the gene (Fig 1a), which is where transcriptional elongation can be the most inefficient [40].

A current model for the role of chromatin in pre-mRNA splicing is that the nucleosome located at an exon acts as a barrier to slow the progress of the elongating RNA Pol II complex, which creates a window of opportunity for splicing factors to execute their splicing function [21,22]. However, as shown here, some gene bodies that are transcribed in the brain and the testis contain H2A.B.3 nucleosomes, which, as discussed above, might facilitate rather than inhibit the progress of an elongating RNA Pol II complex. This argues that a different splicing process might operate in the testis and the brain.

The results presented here show that H2A.B.3 is not a highly expressed variant (representing 3.7% of the total pool of H2A). Consistent with this, it is only found on a subset of active genes and most interestingly, the products of these genes are themselves involved in the processing and function of RNA (S4 Fig) [19]. Given that H2A.B.3 is targeted to a gene concurrent with its activation [3], this raises the possibility that H2A.B.3 may be incorporated into the body of a gene as a result of transcriptional elongation. Also consistent with this notion is that H2A.B.3 is associated with genes that are highly transcribed. Intriguingly though, H2A.B.3 is not incorporated into the +1 nucleosome, a nucleosome that displays a high occupancy but is also proposed to have a high turn over rate [41]. Therefore, simply a higher rate of nucleosome turnover may not be sufficient to incorporate H2A.B.3 suggesting that other, currently unknown, mechanisms may be involved in delivering H2A.B.3 to the body of a gene. Alternatively, other mechanisms may prevent H2A.B.3 from being incorporated into the +1 nucleosome.

**Fig 7:**
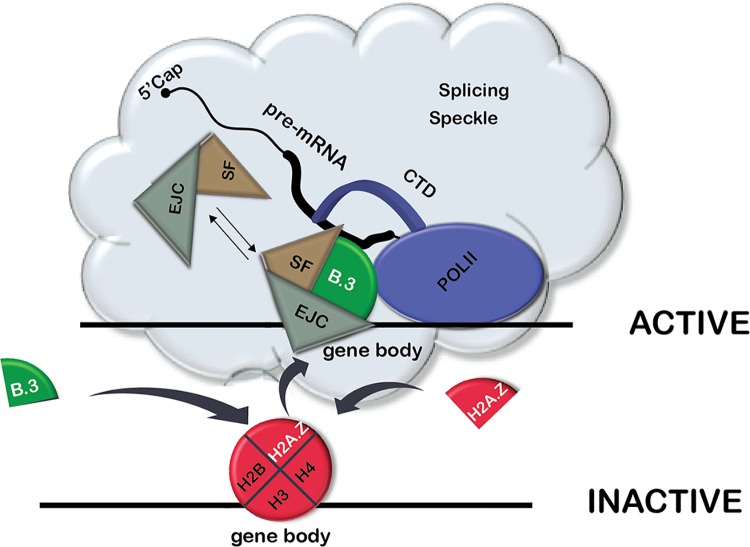
A model depicting the role of H2A.B.3 in recruiting RNA processing factors to the gene body during gene activation. Following the replacement of H2A.Z with H2A.B.3 to assemble active chromatin, H2A.B.3 directly recruits RNA processing factors from splicing speckles to an active gene. Upon transcriptional elongation and the synthesis of mRNA, H2A.B.3 binds and ‘holds’ onto this RNA thus releasing the splicing factors to facilitate the splicing process.

Based on our findings that H2A.B.3: 1) is bound to chromatin (Fig 6b), 2) is located in the body of an active gene (Fig 1), 3) interacts with RNA processing factors (Fig 4, Table 1), 4) binds to RNA, which inhibits its interaction with RNA processing factors and RNA Pol II (Fig 5a, 5c and 5d), 5) associates with mRNA *in vivo* (Fig 5f and 5g), 6) co-localises with splicing speckles (Fig 6) and 7) replaces H2A.Z concurrent with gene activation (Fig 2), we propose the following speculative model (Fig 7). Following the replacement of H2A.Z with H2A.B.3 to assemble active chromatin, H2A.B.3 directly recruits splicing factors from splicing speckles to an active gene. Upon transcriptional elongation and the synthesis of transcript RNA, H2A.B.3 binds and ‘holds’ onto the RNA thus releasing the splicing factors to facilitate the splicing process. In conclusion, H2A.B.3 expands the repertoire of histone functions by being involved in the processing and function of RNA.

## Materials and methods

### Animals

Wild type 28–30 day old male Balb/c mice were used for all testis studies. 6–8 week old mice were used for Hippocampus ChIP and RNA-Seq experiments. Mice were housed according to animal ethics protocol at ANU animal facilities (ANU, Canberra, Australia).

### Immunoprecipitation of germ cell chromatin for mass spectrometry analysis

The preparation of mononucleosomes from seminiferous tubules, as prepared for ChIP-Seq experiments, was carried out as described recently by us. 10–15μg of anti-H2A.B.3 or anti-H2A.Z antibody was covalently bound to magnetic Dynabeads using the Dynabeads antibody coupling Kit (Life Technologies) and incubated with 100–200μg of formaldehyde crosslinked nucleosomes (obtained by MNase I digest, see below) in 50 mM Tris-HCl, pH 7.4, 100 mM NaCl, 1% NP-40, 0.1% SDS; 0.5% sodium deoxycholate, Roche protease inhibitor cocktail for 4 hours at 4°C with rotation. The immunoprecipitated histone variant protein complexes were washed twice in high-salt wash buffer (50 mM Tris-HCl, pH 7.4; 1 M NaCl; 1 mM EDTA; 1% NP-40; 0.1% SDS; 0.5% sodium deoxycholate), and twice with wash buffer (20 mM Tris-HCl, pH 7.4; 10 mM MgCl_2_; 0.2% Tween-20). Beads were resuspended in 20 μl 1x NuPAGE loading buffer (Life Technologies), containing ß-mercaptoethanol, and eluted proteins were loaded on a 4–12% PAGE for electrophoretic separation. The gel was fixed with mass spectrometry-compatible protein stain Instant Blue (Expedeon). Each whole gel lane (H2A.B.3-IP or H2A.Z-IP) was divided in 10–12 segments. The gel segments were dehydrated with acetonitrile (60μl per gel piece) and then dried using a SpeedVac SC100 (Savant). For mass spectrometry analysis, gel slices were destained, reduced and alkylated following the procedure described by Shevchenko *et al*.[42]. Samples were made up to 120 μl using 0.05 M NH_4_HCO_3_, and 40 ng of trypsin (Promega) was then added to each gel slice. Samples were incubated for 16 h at 37°C. Each digest solution was removed to a new microfuge tube and the gel slices treated with the following solutions sequentially for 30 min each: 80 μl 0.1% (v/v) formic acid, 67% (v/v) acetonitrile and 80 μl 100% acetonitrile. The pooled digest and peptide extraction solutions for each sample were then dried (Savant SPD1010, Thermofisher Scientific) before resuspending in 20 μl of 0.1% (v/v) formic acid.

### Mass spectrometry and sequence database searches

Proteolytic peptide samples were separated by nano-LC using an UltiMate 3000 HPLC and autosampler system (Dionex, Amsterdam, Netherlands), and ionized using positive ion mode electrospray following experimental procedures described previously[43]. Single stage mass spectrometry and MS/MS were performed using an LTQ Orbitrap Velos Pro (Thermo Electron, Bremen, Germany) hybrid linear ion trap and Orbitrap mass spectrometer. Survey scans *m*/*z* 350–2000 were acquired in the Orbitrap (resolution = 30 000 at *m*/*z* 400, with an initial accumulation target value of 1,000,000 ions in the linear ion trap; lock mass was applied to polycyclodimethylsiloxane background ions of exact *m/z* 445.1200 and 429.0887). Up to the 15 most abundant ions (>5000 counts) with charge states of >+2 were sequentially isolated and fragmented via collision induced dissociation (CID) with an activation *q* = 0.25, an activation time of 30 ms, normalized collision energy of 30% and at a target value of 10 000 ions; fragment ions were mass analyzed in the linear ion trap. Peak lists derived from LC-MS/MS were generated using Mascot Daemon/ExtractMSn.exe (Matrix Science, Thermo Electron) and submitted to the database search program Mascot (version 2.3, Matrix Science)[44]. The following search parameters were employed: instrument type was ESI-TRAP; precursor ion and peptide fragment mass tolerances were ±5 ppm and ±0.4 Da respectively; variable modifications included were carbamidomethyl (C) and oxidation (M); and enzyme specificity was trypsin with up to 2 missed cleavages. Searches were conducted using the Swiss-Prot database (November 2013 release, 541762 sequence entries); separate searches were conducted against all taxonomies and *Mus musculus* sequences only. Peptide identifications were considered to be high confidence if they were statistically significant (*p*<0.05) according to the Mascot expect metric.

### Immunofluorescence

Hypotonic spreads of male germ cells were prepared essentially as described[45]. Fixed cells were washed in PBS and then blocked for 1 hour with 3% BSA (w/v) in PBS at room temperature. The primary antibody, which was diluted with 1% BSA (w/v), 0.1% Tween 20 (v/v) in PBS were applied to slides and incubated for 16 hours at 4°C in a humidity chamber. Following three washes with PBS, slides were incubated with fluorophore-conjugated secondary antibodies for 1 hour at room temperature. Following a further three washes with PBS, slides were incubated in 1 μM DAPI for 2 min. Vectashild (Vecta laboratories) was applied to prevent slides from photo bleaching.

### Round spermatid isolation and fractionation

Round spermatids were isolated by sedimentation on a BSA gradient as described elsewhere [46]. Subcellular fractionation of round spermatids was carried out in LSBD buffer (50mM Hepes, pH 7; 3mM MgCl2; 20% glycerol (v/v); 1% NP40 (v/v) plus 250 mM KCl to obtain the cytoplasmic and nucleoplasmic fraction or 500mM KCl to obtain the fraction containing loosely bound chromatin proteins. The remaining material was designated as the chromatin fraction. Prior to immunoprecipitation with affinity purified H2A.B.3 antibodies, the chromatin fraction was sonicated and treated with benzonase (Millipore).

### Chromatin preparation for ChIP

Mononucleosomes were prepared from testicular tubules using Micrococcal nuclease I (Mnase I) digestion as described [3]. To prepare chromatin from hippocampal tissues, 10 Balb/c male mice (6–10 weeks old) were decapitated and hippocampi were surgically removed into ice cold Hank's Balanced Salt Solution (HBSS, Sigma) buffered with 50mM Hepes pH-7.6 and supplemented with 0.2 mM PMSF and EDTA-free protein inhibitor cocktail (Roche)). Hippocampal slices were homogenised in a Dounce homogeniser with 5–10 strokes using pestle A with 4 ml of ice-cold HBSS. Cells were counted and 2–5 x 10^7^ cells were fixed for 12 min with rotation at room temperature in 10 ml of fresh medium HBSS in the presence of 1.2% (v/v) formaldehyde. Mononucleosomes were obtained by digesting purified nuclei with 2–4 units of MNase I (NEB) at 37C.

#### ChIP assays, preparation of ChIP—Seq libraries, and DNA sequencing analysis

Chromatin immunoprecipitations were performed with affinity purified H2A.B.3, H2A.Z and H3K36me3 antibodies (5 μg, see below) as described by us previously. Two biological replicates were performed for each ChIP-Seq experiment. Libraries were prepared for the first biological replicate using the Sample Prep Kit (Illumina) according to the manufacturer's protocol for single end sequencing of DNA. The second biological replicate used the ChIP-Seq Library Prep (New England Biolabs) with barcodes and paired end adaptors. Quality and concentration of the libraries were assessed on Agilent Technologies 2100 Bioanalyzer and using qPCR with adaptor specific primers (ABI Prism 7900HT) according to Illumina's recommendations. DNA was sequenced on HiSeq 2000 or HiSeq 2500 (rapid run mode) (Illumina) using 100 base pairs single—or paired—end reads. Mapped reads for all input and ChIP-Seq experiments (and RNA-Seq plus RNA-IP Seq experiments) are shown in S4 Table. Both biological replicates for H2A.B.3, H2A.Z and H3K36me3 yielded the same observations and therefore the analysis of the paired-end reads are shown here. To perform quantitative H2A.B.3 or H2A.Z ChIP assays on specific genes, ChIP DNA libraries were prepared in triplicate as described above. The DNA concentration of each individual library was measured by real-time qPCR (ABI Prism 7900HT) using Power SYBR Green PCR master mix and standard settings (Applied Biosystems) with adaptor-specific primers (Illumina). Each ChIP DNA library was adjusted accordingly to the same concentration and then used for qPCR (80 pM final concentration) with specific primers (primers sequences are listed in Additional File 1; S3 Table). The relative sample enrichment was calculated with the following formula: 2^-ΔΔCt^.

#### RNA-IP Seq

The RNA-IP procedure to detect H2A.B.3 associated RNA was carried out as described [47] with some modifications. In particular, we found that cells UV-irradiated with 150mJ/cm^2^ at 254 nm produced a high background of non-specific protein-RNA interactions masking specific protein-RNA interactions. We therefore reduced the irradiation to 75mJ/cm^2^ at 254 nm. A single cell suspension prepared from testicular tubules was washed 3 times with cold DPBS-GL (DPBS (Gibco) supplemented with 5.6mM D-glucose, 5.4mM DL-lactate, 0.5mM DTT, 0.2mM PMSF, EDTA-free protein inhibitor cocktail (Roche), pH-7.1). These cells were then resuspended in 6 ml at a density of 4-6x10^6^ cells per ml in the same buffer and UV-irradiated with 75mJ/cm^2^ at 254 nm (Stratalinker 1800, Stratagene). Cells were then cross-linked with 1.2% (v/v) formaldehyde. To prepare chromatin for RNA-IP-seq, MNase I digestion was substituted by sonication (since this enzymes digests also RNA). Purified nuclei from germ cells were re-suspended in 2ml of sonication buffer (50mM Hepes pH-7.6, 1mMEDTA, 05mM EGTA, 0.1% (w/v) SDS, 0.1% (w/v) sodium deoxycholate, 0.2 mM PMSF, Roche EDTA-free protein inhibitor cocktail) and sonicated for 15 minutes (30 seconds intervals on/off at high settings) at 4^°^C using a Bioraptor Sonicator (Diagenode) to obtain ~ 300 base pair-long chromatin fragments. After sonication, the sheared chromatin was cleared by centrifugation at 10000 g for 5 minutes, dialysed against 10 mM Tris-HCl pH 7.6, 1 mM EDTA, 0.5 mM EGTA, 4% (v/v) glycerol and stored at -80°C. 300μg of sheared testis chromatin was used for the RNA-IP procedure to detect H2A.B.3 associated RNA [47] with the following modification. To minimize non-specific RNA carryover by eluted IgGs, 20μg of antibody was covalently linked to dynabeads (Life Technologies). As a control, we also performed this procedure using affinity purified H2A.Z antibodies. Only RNA-protein complexes that were not present in chromatin immunopurified with H2A.Z were chosen, and combined, for further analysis. RNA-IP libraries were prepared and sequenced using 100 base pair paired—end reads. Our analyses also involved subtracting normalized H2A.Z CLIP RNA reads from normalized H2A.B.3 CLIP RNA reads at each base position genome-wide.

### RNA-Seq libraries

Total RNA was isolated using TRIzol (Invitrogen) and the RNeasy Mini kit (Qiagen). Samples were treated with TURBO^™^ DNase (Life Technologies). RNA-Seq libraries (three biological replicates) were prepared using NEBNext mRNA Library Prep kit using oligo-dT enrichment module (New England Biolabs) following manufacturer’s recommendations. Resulted RNA—Seq libraries were sequenced on HiSeq 2000 sequencer (Illumina) using 100 base pairs paired-end reads.

### RNase I treatment of germ cell lysates and CLIP

Total germ cells from 28–30 day mice testis were UV-irradiated once with 75mJ/cm^2^ at 254 nm to crosslink RNA/protein complexes in a UV-irradiation oven, Stratalinker 1800, (Stratagene). 3-4x10^7^cells were lysed in 2 ml of lysis buffer (50 mM Tris-HCl pH 7.6; 100 mM NaCl; 5mM MgCl_2_; 1% NP-40, 1mM DTT, Roche protease inhibitor cocktail, pH 7.4) for 30 min on ice. Chromatin was mechanically sheared by passing nuclei through a 31G syringe. To remove insoluble material, the lysates were centrifuged at 10,000 g. Half of the supernatant was subjected to RNase I treatment (800 units, Ambion AM2294) for 30 min at 37°C, while the other half was treated in the same way but without the addition of RNase I. The degradation of RNA was monitored by the Qubit HS RNA assay. Both untreated and RNAase I-treated lysates were immunoprecipitated with H2A.B.3 antibodies exactly as described for IP-MS.

To distinguish IPs performed with formalin and UV-crosslinked cells from UV-only-crosslinked cells, we use the term CLIP (UV-cross-linking immuno precipitation) for the latter samples only. For CLIP assays, germ cell lysates were prepared in an identical manner as just described. 0.25ml of lysate was diluted in 1 ml of 50mM Tris, 100mM NaCl, 5mM MgCl2, 1mM DTT for RNase treatement with 400 units of RNase I (Ambion AM2294) for 30 min at 37°C. Treated lysates were immunoprecipitated with equal amount of anti-H2A.B.3 and anti-H2A.Z antibodies (10μg each), bound to protein A/G dynabeads (Thermo Fisher), for 2 hours. Following washing, twice with high salt wash buffer (50 mm Tris-HCl; 1M NaCl; 5mM MgCl_2_; 1% NP-40, 0.1% SDS, 0.5% Na deoxycholate, pH 7.4) and twice with wash buffer (20mM Tris-HCL, 10mM MgCL2, 0.2% Tween-20, pH 7.4)), immunoprecipitated complexes were dephosphorylated on beads using PNK and than the RNA was 5' end labeled with P^32^ gamma-ATP. Eluted labeled complexes were electrophoresed through SDS-PAGE and transferred onto a nitrocellulose membrane. The same membrane was uses for a western analysis using anti-H2A.B3 and anti-H2AZ antibodies.

### Electrophoretic mobility RNA gel-shift assays

Histone H2A-H2B (and variant) dimers were produced using standard protocols for refolding of histone complexes [48]. Either a 222 nt or 152 nt RNA was *in vitro* transcribed from the pcDNA 3.1 linearised plasmid template containing the RNA probe of interest [35] using a HiScribe T7 Quick High Yield RNA Synthesis Kit (New England Biolabs). The RNA probe was heated at 95°C for 5 min, then rapidly cooled on ice for 2 min just prior to setting up binding experiments. The RNA (20 ng) was then incubated on ice with the relevant histone dimers in binding buffer (10 mM MOPS, pH 7.5, 200 mM NaCl, 5 mM MgCl_2_, 10% glycerol (v/v), 1 mM DTT, 0.03 mg/ml heparin). The binding reactions were analysed on 5% acrylamide 1x TB gels. Gels were stained with SYBR Gold Nucleic Acid Gel Stain and visualised using a Typhoon FLA 9000. Biotinylated peptides corresponding to the N-terminal tails of histones H2A, H2A.Z, H2A.B, and H2A.B.3 were purchased from GL Biochem at >90% purity. A Cy3-labelled 25 nt RNA (Cy3-CAGCGACUCGGGUUAUGUGAUGGAC) was purchased from Sigma-Aldrich. The biotinylated peptides (30 pmol) were immobilised on steptavidin-coated M-280 Dynabeads (LifeTechnologies) and incubated with 150 pmol of the Cy3-labelled RNA in binding buffer for 1 hr at room temperature. The beads were then washed three times with binding buffer. Following the final wash, the beads were resuspended in Novex TBE-urea Sample Buffer (Life Technologies), heated at 70°C for 2 min and then run on 15% Novex TBE-urea gels (Life Technologies). Cy3 fluorescence was visualised on a Typhoon FLA 9000.

### Antibodies

Antibodies used were as follows. Anti-H2A.B.3[3], Anti-H2A.Z [49], Anti-H3K36me3 (ab9050), Anti-Smith Antigen [Y12] (ab3138); Anti-H2A (ab18255), anti-H3 (ab1791), anti-RNA PolII (phospho S2) (ab5095), anti-RNA PolII (phospho S5) (ab5131), anti-Rent1 (ab109363), anti-Snrpa1 (ab128937), anti-U1A (ab155054), anti-SPT6 (ab32820), anti-Symplekin (ab80274), anti-SAP18 (ab31748) and anti-Sf3b155 (ab66774), all from Abcam. Anti-DDX39A (PA5-31220, Pierce), anti-sheep-HRP (AP324R; Chemicon), anti-rabbit-HRP (AP322P; Chemicon).

### Bioinformatic analyses

Chip-Seq reads were adaptor trimmed and mapped to the genome using Bowtie2[50]. Paired end reads were converted to single spanning fragments. Coverage bedGraphs were generated. Plots of the mean coverage anchored at genomic landmarks (the intron—exon boundaryor the TSS) for a span of +1000 to -1000 base pairs by aligning local coverage at these landmarks genome-wide using UCSC genes canonical transcript annotations. Units of RPM (mean reads per base pair per million reads mapped) were used normalised by the total mapped library size. Metagenes plots were generated by scaling exons to the same normalised x-axis from 0 to 1. Intron—exon boundaries that are alternatively spliced in mouse hippocampus and testes were determined from paired-end RNA-Seq, which was adaptor, trimmed by Trimmomatic[51] in palindromic mode and mapped using Bowtie 2. Alternatively spliced exons were called using the MATS software[52]; a constitutive set was formed by removing alternative sites that overlapped the UCSC annotations. To test whether H2A.B.3 coverage over exons was correlated with alternative or constitutive splicing, the mean RPM H2A.B.3 coverage was compared over the exons (bases 0 to 150 downstream of the intron-exon junction) of alternatively spliced versus and constitutively spliced axons. To ensure the sets were comparable and not confounded by possible biases toward a higher expression level for the alternative spliced set, the larger constitutive set was subsampled to the same size as the alternative spliced set such that it had a matched distribution of gene expression levels. Alternatively spliced exons were called using the Multivariate Analysis of Transcript Splicing (MATS) software. This was applied to stranded RNA-seq data (three replicates per tissue). Skipped "cassette" exons were identified to define AS sites. A constitutive set was formed by removing these alternative sites from the full set of UCSC intron-exon annotations. Inclusion fraction relative to the flanking constitutive exons was estimated for each cassette exon by the MATS software.

RNA-IP data was mapped with Tophat [53] using bowtie2. Adapters were trimmed using Trimmomatic. To compare correlations between two ChIP-Seq data sets relative to the intron to exon boundary, we performed Pearson correlation between the vectors of mean coverage formed over a fixed window of size 50 base pairs at a given distance from the intron—exon anchor. To determine if exons versus introns gained H2A.B.3 preferentially, the association between mean H2A.B.3/input ratio (in RPM) and log expression from RNA-Seq data (RPM) was calculated for a 1kb flanking window around all intron-exon boundaries. At each base position relative to the intron exon-boundary, a linear model was fitted to the H2A.B.3/input ratio versus gene log expression and the gradient of the fitted model plotted (using the mean H2A.B.3/input ratio value in a sliding 20 bp window around each bp).

To determine if exons versus introns gained H2A.B.3 preferentially, the association between mean H2A.B.3/input ratio (in RPM) and log expression from RNA-Seq data (RPM) was calculated for a 1kb flanking window around all intron-exon boundaries. At each base position relative to the intron-exon boundary, a linear model was fit to the H2A.B.3/input ratio versus gene log expression, using the mean H2A.B.3/input ratio value in a sliding 20 bp window around each bp, and the slope of the fitted model plotted. When overlapping RNA-IP data with H2A.B.3 ChIP-Seq data over gene bodies and promoters (-1000 bp upstream of TSS), a minimum coverage of 30 reads per base was used, which was above background reads. To determine the percentage of H2A.B.3 expression compared to the total H2A pool, the mean TPM was determined for all different H2A subtype genes expressed.

## Supporting information

S1 Fig A positive and negative correlation between the incorporation of H2A.B.3 and H2A.Z at the intron—exon boundary and gene expression, respectively. (a) The normalised distribution of input (0 to 0.15 RPM) at the intron—exon boundary for all genes separated into 100 groups in the testis as a heat map. (b) The normalised distribution of H2A.B.3 (0 to 0.15 RPM) at the intron—exon boundary for all genes separated into 100 groups in the testis as a heat map. (c) The normalised distribution of H3K36me3 (0 to 0.15 RPM) at the intron—exon boundary for all genes separated into 100 groups in the testis as a heat map. (d) The normalised distribution of H2A.Z (0 to 0.15 RPM) at the intron—exon boundary for all genes separated into 100 groups in the testis as a heat map.
(PDF)

S2 Fig The H2A.B.3 nucleosome is located closer to the exon-intron boundary than to the intron-exon boundary. (a) A testis H2A.B.3 meta-intron plot where the length of all introns are normalised to the same size. (b) A testis H2A.B.3 meta-exon plot where the length of all exons are normalised to the same size. (c) The individual line represents the normalised H2A.B.3 reads aligned between -1 and +1 kb from the exon—intron boundary for all exons in the testis.
(PDF)

S3 Fig Mouse histone variants H2A.B.3 and H2A.Z are expressed in hippocampal neurons. Paraffin-embedded mouse brain sections were indirectly immunostained with H2A.B.3 and H2A.Z antibodies. The primary antibody signal was amplified with the TSA signal amplification system (Perkin Elmer). Representative images of the hippocampus and surrounding regions are shown.
(PDF)

S4 Fig H2A.B.3 is located at the TSS of active genes in both the testis and brain. (a) Normalised testis H2A.B.3 ChIP-Seq reads (mean reads per base pair per million reads mapped (RPM)) aligned between -1 and +1 kb from the TSS ranked according to their level of expression. (b) Normalised hippocampus H2A.B.3 ChIP-Seq reads ranked according to their level of expression aligned with the TSS. (c) Normalised testis input Seq reads aligned between -1 and +1 kb from the TSS ranked according to their level of expression. (d) Normalised hippocampus input Seq reads ranked according to their level of expression aligned with the TSS. (e) The normalised distribution of H2A.B.3 (0 to 0.15 RPM) at the TSS for all genes separated into 100 groups in the testis as a heat map. (f) The normalised distribution of H2A.B.3 (0 to 0.15 RPM) at the TSS for all genes separated into 100 groups in the hippocampus as a heat map. To detect localised biological functions over nucleosomes in the H2A.B.3 ChIP-Seq and input data sets, gene set enrichment analyses were performed of gene symbols ranked by the mean coverage over a fixed window size of 50 bp at successive distances from the TSS. This enrichment was computed for H2A.B.3 and input using gene sets corresponding to GO terms for the testis (g) and the hippocampus (h). Shown are those GO terms where H2A.B.3 was most highly enriched.
(PDF)

S5 Fig H2A.B.3 is located at the intron—exon boundary of active genes in the hippocampus. Input nucleosomes, nucleosomes immunoprecipitated with H2A.B.3 or H3K36me3 affinity purified antibodies, and poly (A)-transcripts obtained from adult hippocampal neurons were sequenced yielding 100 base pair paired-end reads. (a) The individual lines represent the normalised H2A.B.3 and total input reads aligned between -1 and +10 kb from the TSS in the hippocampus. (b) The individual line represents the normalized input nucleosome reads (mean reads per base pair per million reads mapped (RPM)) aligned between -1 and +1 kb from the intron—exon boundary for all exons in the hippocampus ranked according their expression level (repressed, low, medium and high). The colour-map panel shows the relationship between colour and the gene expression rank. (c) Normalized hippocampus H2A.B.3 ChIP-Seq reads ranked according to their expression level aligned with the intron—exon boundary. (d) At each base position relative to the intron exon-boundary, a linear model was fitted to the mean H2A.B.3/input ratio versus gene log expression across all intron—exon boundaries, and the slope of the fitted model plotted for the hippocampus. (e) Normalized hippocampus H3K36me3 ChIP-Seq reads ranked according to their expression level aligned with the intron—exon boundary. **(f)** Pearson correlation of the log coverage, across 50 base pair windows, was calculated between hippocampus H2A.B.3 ChIP-Seq reads and H3K36me3 ChIP-Seq reads for each base pair relative to the intron—exon boundary. (g) Normalized hippocampus H3K36me3 ChIP-Seq reads ranked according to the incorporation of H2A.B.3 (very low, low, medium and high) aligned with the intron—exon boundary. 95% confidence bands are shown in grey.
(PDF)

S6 Fig A direct link between the incorporation of H2A.B.3 at the TSS and the intron—exon boundary. (a) Normalised testis H2A.B.3 ChIP-Seq reads ranked according to the incorporation of H2A.B.3 at the TSS (very low, low, medium and high) aligned with the intron—exon boundary. (b) Normalised hippocampus H2A.B.3 ChIP-Seq reads ranked according to the incorporation of H2A.B.3 at the TSS (very low, low, medium and high) aligned with the intron—exon boundary.
(PDF)

S7 Fig H2A.B.3 is located at the intron—exon boundary of constitutive exons. (a) Normalized testis H2A.B.3 ChIP-Seq reads ranked according to their expression level aligned with the intron—exon boundary of constitutive exons. (b) Normalized hippocampus H2A.B.3 ChIP-Seq reads ranked according to their expression level aligned with the intron—exon boundary of constitutive exons.
(PDF)

S8 Fig Relative expression of H2A.B.3 and H2A.Z at different stages of mouse spermatogenesis. Total RNA was extracted from whole mouse testes at various stages of spermatogenesis. cDNA was synthesised using random and poly(dT) priming. Comparative C_T_ method was used to calculate relative gene expression; mouse β-actin was used as an endogenous reference gene. 10do: 10 day old testis (all cell types prior to meiosis); 12do: 12 day old testis (all cell types up to early leptotene); 19do: 19 day old testis (all cell types up to late pachytene); 24do: 24 day old testis (all cell types up to early round spermatids), 28do: 28 day old testis (all stages up to late round spermatids), 65do: adult mice testis.
(PDF)

S9 Fig H2A.B.3 does not associated with RNA at the TSS in the testis. Following the RNA—H2A.B.3 IP procedure, the released RNA was sequenced to yield 100 base pair paired end reads. A H2A.B.3 RNA plot ranked according to expression aligned with the TSS.
(PDF)

S1 Table Other proteins that co-immunoprecipitate with H2A.B.3 but not with H2A.Z identified by Mass Spectrometry as described in Table 1. (PDF)

S2 Table Histone proteins that co-immunoprecipitate with H2A.B.3 identified by Mass Spectrometry as described in Table 1. (PDF)

S3 Table Real-time PCR primer sequences for ChIP assays used in the study. (PDF)

S4 Table List of lllumina libraries used in this study. (PDF)
