# Supplementary figures and images for: A new link between transcriptional initiation and pre-mRNA splicing: The RNA binding histone variant H2A.B
Source: PLoS Genet. 2017 Feb 24;13(2):e1006633. doi: 10.1371/journal.pgen.1006633 (PMC5345878; doi:10.1371/journal.pgen.1006633)

# Supporting Figure 1

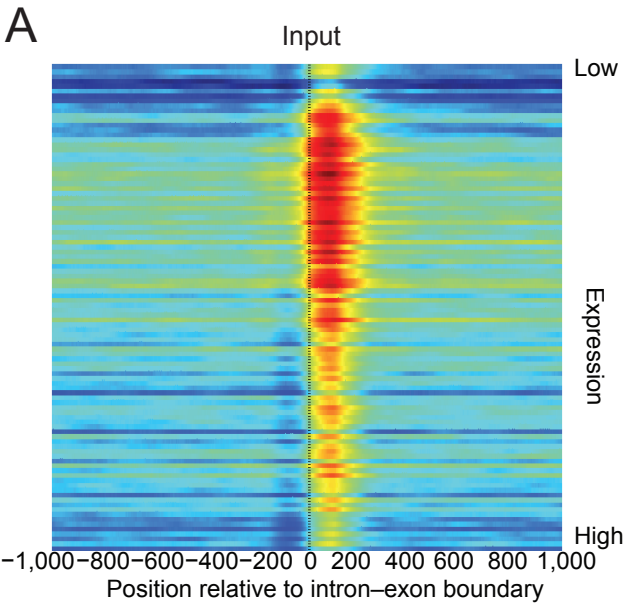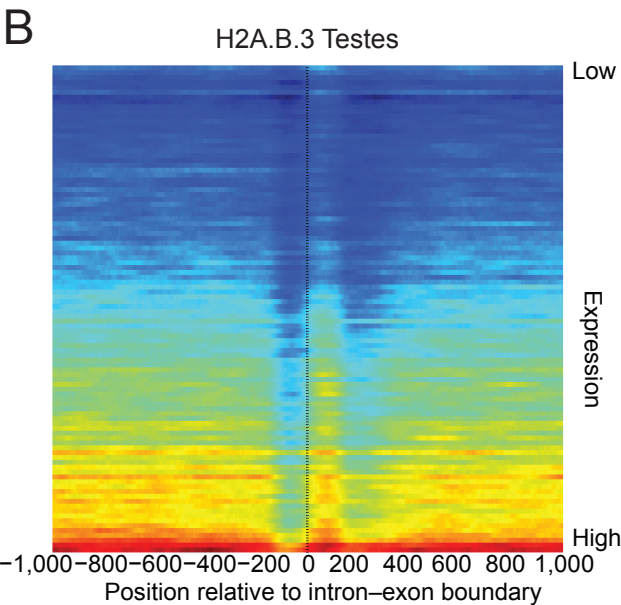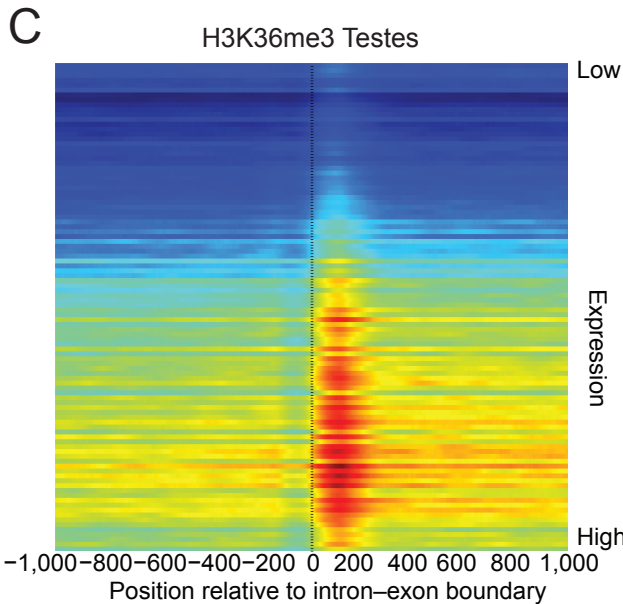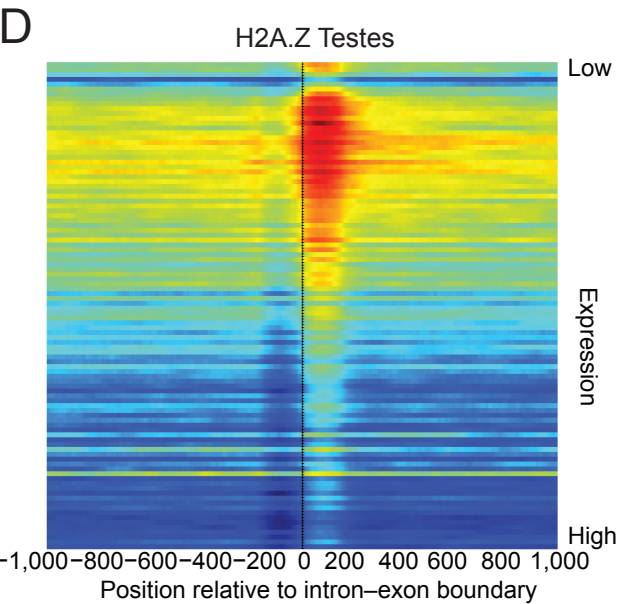

Supplement: S1 Fig — (a) The normalised distribution of input (0 to 0.15 RPM) at the intron—exon boundary for all genes separated into 100 groups in the testis as a heat map. (b) The normalised distribution of H2A.B.3 (0 to 0.15 RPM) at the intron—exon boundary for all genes separated into 100 groups in the testis as a heat map. (c) The normalised distribution of H3K36me3 (0 to 0.15 RPM) at the intron—exon boundary for all genes separated into 100 groups in the testis as a heat map. (d) The normalised distribution of H2A.Z (0 to 0.15 RPM) at the intron—exon boundary for all genes separated into 100 groups in the testis as a heat map. (PDF) [file pgen.1006633.s001.pdf]

# Supporting Figure 2

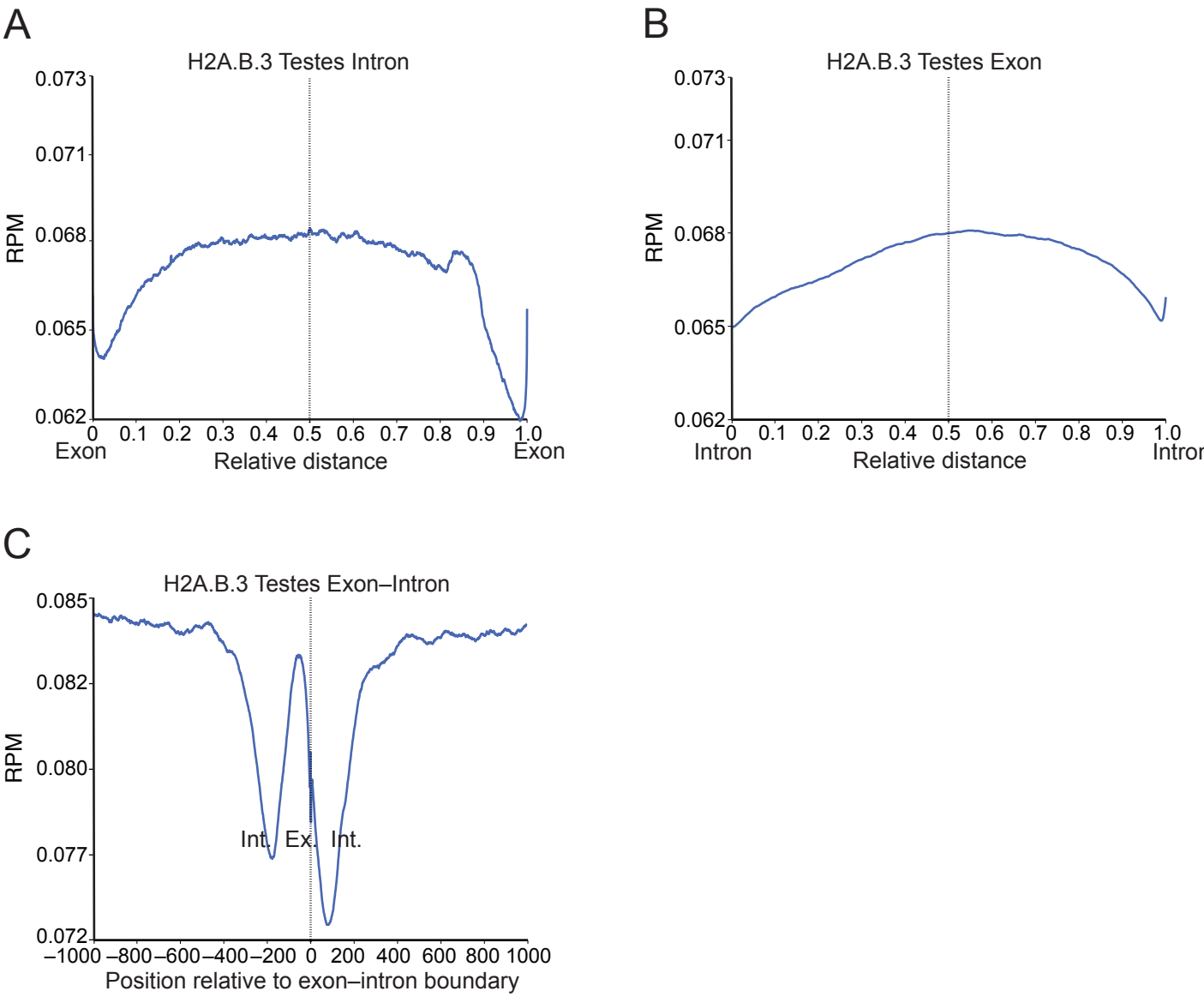

Supplement: S2 Fig — (a) A testis H2A.B.3 meta-intron plot where the length of all introns are normalised to the same size. (b) A testis H2A.B.3 meta-exon plot where the length of all exons are normalised to the same size. (c) The individual line represents the normalised H2A.B.3 reads aligned between -1 and +1 kb from the exon—intron boundary for all exons in the testis. (PDF) [file pgen.1006633.s002.pdf]

## Supporting Figure 3

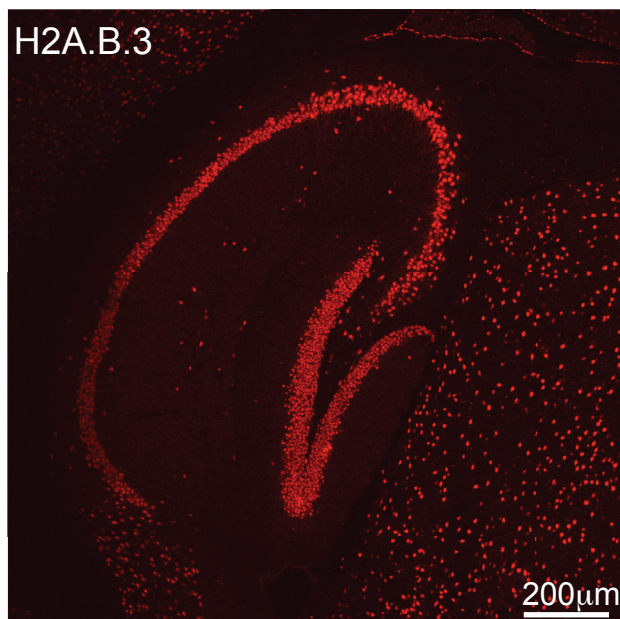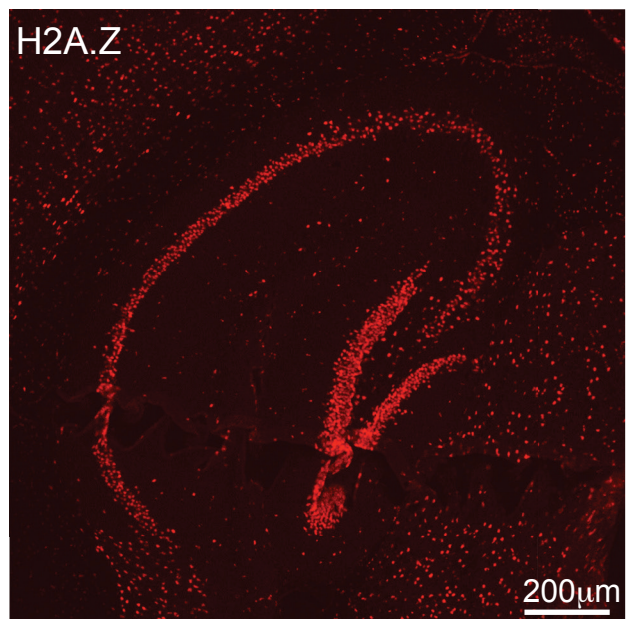

Supplement: S3 Fig — Paraffin-embedded mouse brain sections were indirectly immunostained with H2A.B.3 and H2A.Z antibodies. The primary antibody signal was amplified with the TSA signal amplification system (Perkin Elmer). Representative images of the hippocampus and surrounding regions are shown. (PDF) [file pgen.1006633.s003.pdf]

Supporting Figure 4

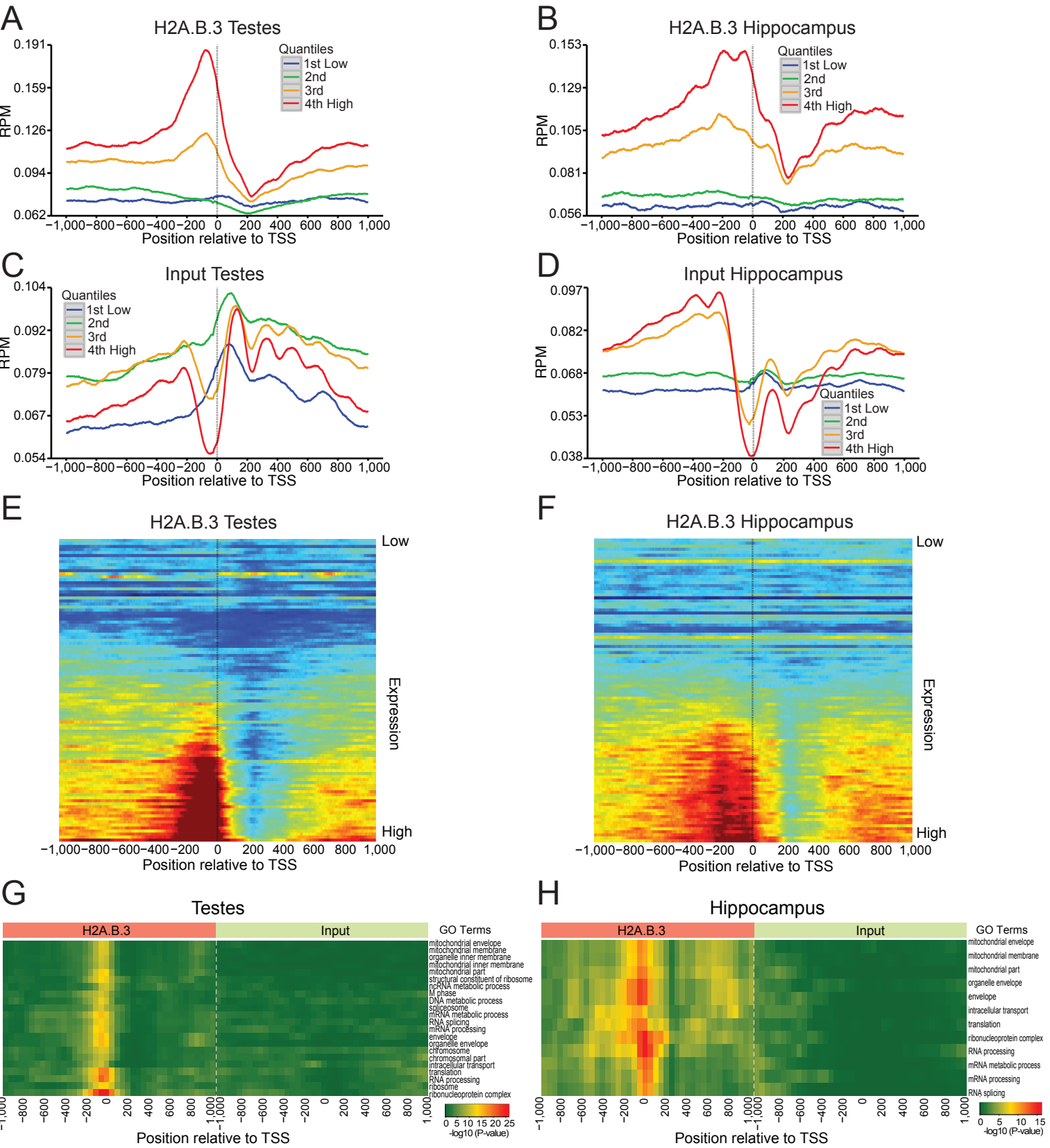

Supplement: S4 Fig — (a) Normalised testis H2A.B.3 ChIP-Seq reads (mean reads per base pair per million reads mapped (RPM)) aligned between -1 and +1 kb from the TSS ranked according to their level of expression. (b) Normalised hippocampus H2A.B.3 ChIP-Seq reads ranked according to their level of expression aligned with the TSS. (c) Normalised testis input Seq reads aligned between -1 and +1 kb from the TSS ranked according to their level of expression. (d) Normalised hippocampus input Seq reads ranked according to their level of expression aligned with the TSS. (e) The normalised distribution of H2A.B.3 (0 to 0.15 RPM) at the TSS for all genes separated into 100 groups in the testis as a heat map. (f) The normalised distribution of H2A.B.3 (0 to 0.15 RPM) at the TSS for all genes separated into 100 groups in the hippocampus as a heat map. To detect localised biological functions over nucleosomes in the H2A.B.3 ChIP-Seq and input data sets, gene set enrichment analyses were performed of gene symbols ranked by the mean coverage over a fixed window size of 50 bp at successive distances from the TSS. This enrichment was computed for H2A.B.3 and input using gene sets corresponding to GO terms for the testis (g) and the hippocampus (h). Shown are those GO terms where H2A.B.3 was most highly enriched. (PDF) [file pgen.1006633.s004.pdf]

# Supporting Figure 5

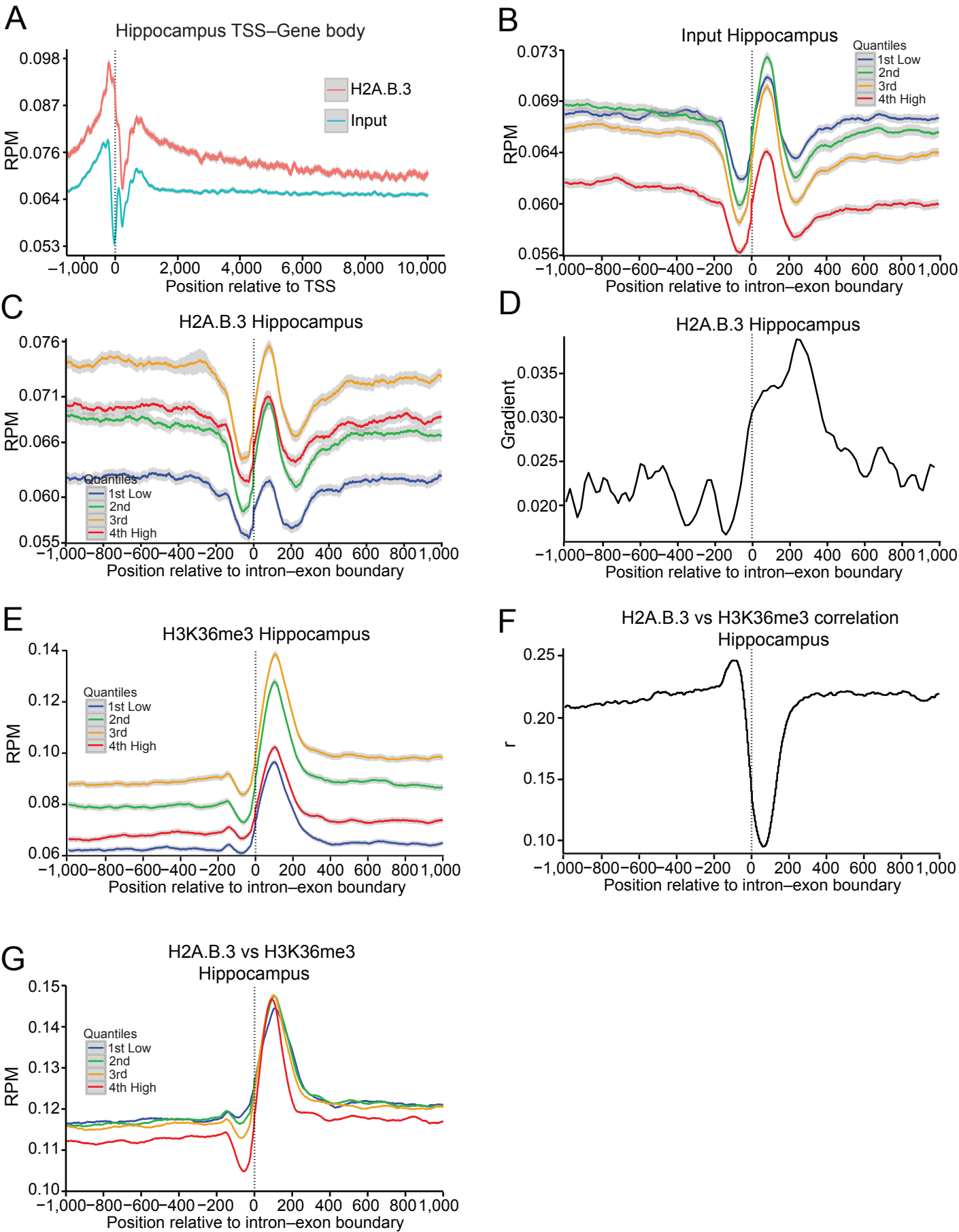

Supplement: S5 Fig — Input nucleosomes, nucleosomes immunoprecipitated with H2A.B.3 or H3K36me3 affinity purified antibodies, and poly (A)-transcripts obtained from adult hippocampal neurons were sequenced yielding 100 base pair paired-end reads. (a) The individual lines represent the normalised H2A.B.3 and total input reads aligned between -1 and +10 kb from the TSS in the hippocampus. (b) The individual line represents the normalized input nucleosome reads (mean reads per base pair per million reads mapped (RPM)) aligned between -1 and +1 kb from the intron—exon boundary for all exons in the hippocampus ranked according their expression level (repressed, low, medium and high). The colour-map panel shows the relationship between colour and the gene expression rank. (c) Normalized hippocampus H2A.B.3 ChIP-Seq reads ranked according to their expression level aligned with the intron—exon boundary. (d) At each base position relative to the intron exon-boundary, a linear model was fitted to the mean H2A.B.3/input ratio versus gene log expression across all intron—exon boundaries, and the slope of the fitted model plotted for the hippocampus. (e) Normalized hippocampus H3K36me3 ChIP-Seq reads ranked according to their expression level aligned with the intron—exon boundary. (f) Pearson correlation of the log coverage, across 50 base pair windows, was calculated between hippocampus H2A.B.3 ChIP-Seq reads and H3K36me3 ChIP-Seq reads for each base pair relative to the intron—exon boundary. (g) Normalized hippocampus H3K36me3 ChIP-Seq reads ranked according to the incorporation of H2A.B.3 (very low, low, medium and high) aligned with the intron—exon boundary. 95% confidence bands are shown in grey. (PDF) [file pgen.1006633.s005.pdf]

# Supporting Figure 6

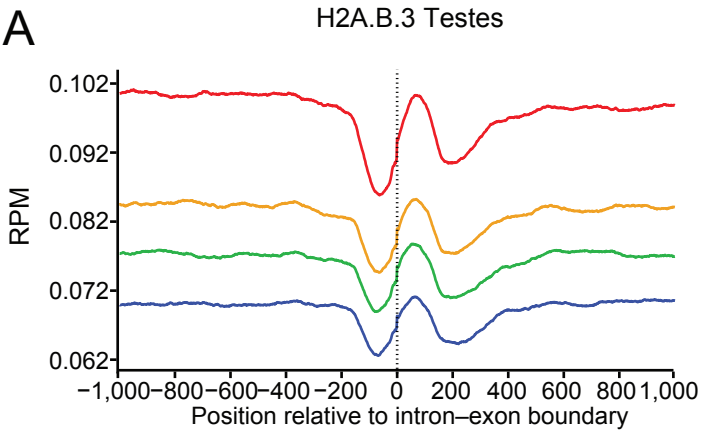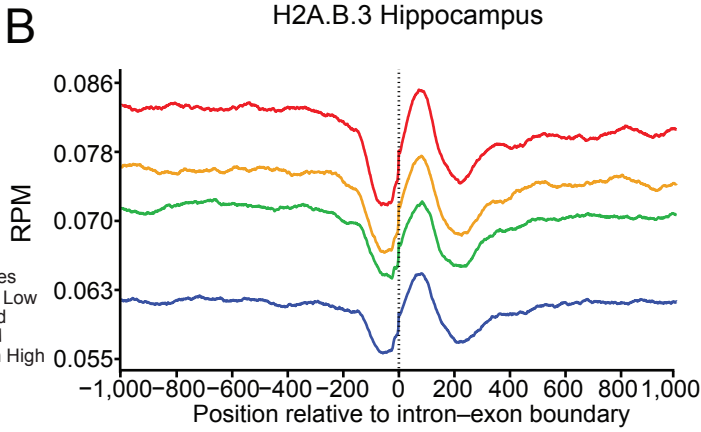

Supplement: S6 Fig — (a) Normalised testis H2A.B.3 ChIP-Seq reads ranked according to the incorporation of H2A.B.3 at the TSS (very low, low, medium and high) aligned with the intron—exon boundary. (b) Normalised hippocampus H2A.B.3 ChIP-Seq reads ranked according to the incorporation of H2A.B.3 at the TSS (very low, low, medium and high) aligned with the intron—exon boundary. (PDF) [file pgen.1006633.s006.pdf]

# Supporting Figure 7

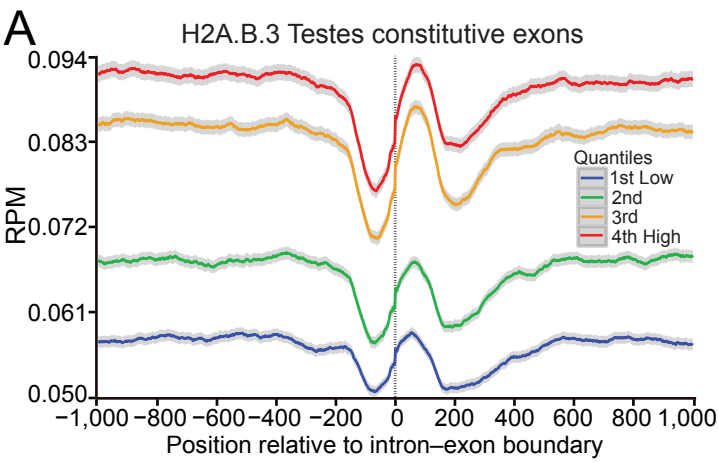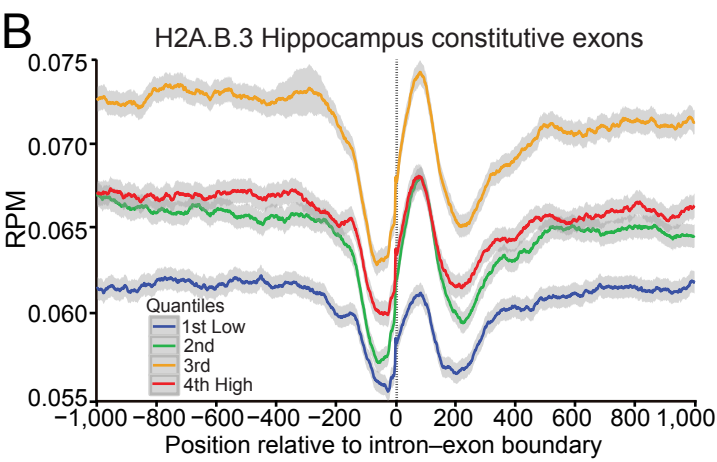

Supplement: S7 Fig — (a) Normalized testis H2A.B.3 ChIP-Seq reads ranked according to their expression level aligned with the intron—exon boundary of constitutive exons. (b) Normalized hippocampus H2A.B.3 ChIP-Seq reads ranked according to their expression level aligned with the intron—exon boundary of constitutive exons. (PDF) [file pgen.1006633.s007.pdf]

Supporting Figure 8

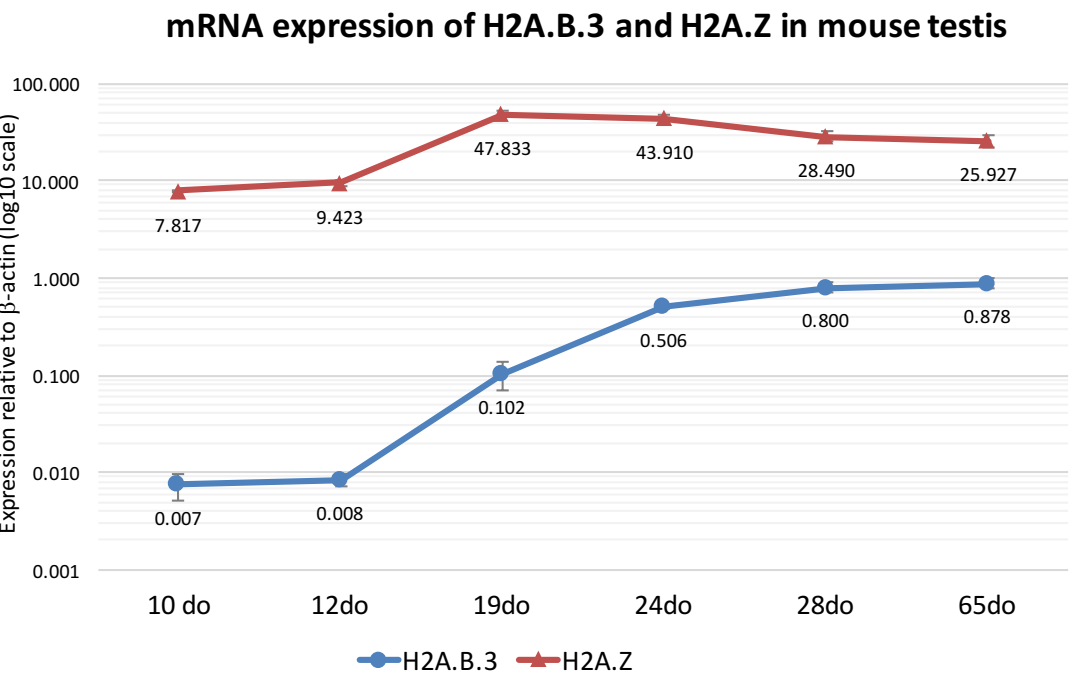

Supplement: S8 Fig — Total RNA was extracted from whole mouse testes at various stages of spermatogenesis. cDNA was synthesised using random and poly(dT) priming. Comparative CT method was used to calculate relative gene expression; mouse β-actin was used as an endogenous reference gene. 10do: 10 day old testis (all cell types prior to meiosis); 12do: 12 day old testis (all cell types up to early leptotene); 19do: 19 day old testis (all cell types up to late pachytene); 24do: 24 day old testis (all cell types up to early round spermatids), 28do: 28 day old testis (all stages up to late round spermatids), 65do: adult mice testis. (PDF) [file pgen.1006633.s008.pdf]

# Supporting Figure 9

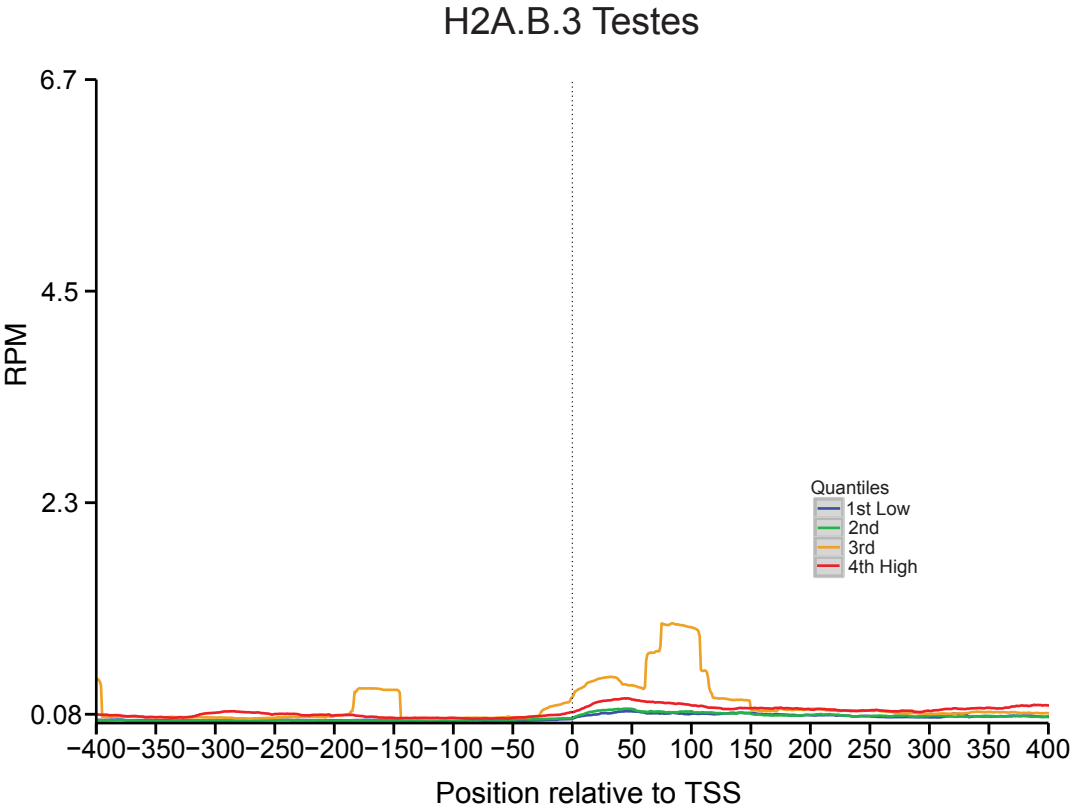

Supplement: S9 Fig — Following the RNA—H2A.B.3 IP procedure, the released RNA was sequenced to yield 100 base pair paired end reads. A H2A.B.3 RNA plot ranked according to expression aligned with the TSS. (PDF) [file pgen.1006633.s009.pdf]
