# Supplementary material for: A new link between transcriptional initiation and pre-mRNA splicing: The RNA binding histone variant H2A.B
Source: PLoS Genet. 2017 Feb 24;13(2):e1006633. doi: 10.1371/journal.pgen.1006633 (PMC5345878; doi:10.1371/journal.pgen.1006633)
Supplement: S1 Table — (PDF) [file pgen.1006633.s010.pdf]

Supporting Table 1

Other proteins that co-immunoprecipitate with H2A.B.3 but not with H2A.Z identified by Mass Spectrometry as described in Table 1

ACCESSION ID	PROTEIN NAME	GENE NAME	UNIQUE PEPTIDES #	MW (KDA)	PROCESS
			NOT GROUPED		
P17742	Peptidyl-prolyl cis-trans isomerase A	Ppia	2	18	Folding of proteins
Q3UKC1	Tax1-binding protein 1	Tax1bp1	2	93.6	Inhibits TNF-induced apoptosis
Q61464	Zinc finger protein 638	Znf638	2	218	Early regulator of adipogenesis
Q80UG5	Septin-9	Sept9	2	65.5	Cytokinesis /cell division
P42208	Septin-2	Sept2	4	41.5	Cytokinesis/cell division
Q9D1R9	60S ribosomal protein L34	Rpl34	2	13.3	A component of the 60S subunit of the ribosome
P62908	40S ribosomal protein S3	Rps3	3	26.7	A component of the 40S subunit of the ribosome
