# Supplementary material for: A new link between transcriptional initiation and pre-mRNA splicing: The RNA binding histone variant H2A.B
Source: PLoS Genet. 2017 Feb 24;13(2):e1006633. doi: 10.1371/journal.pgen.1006633 (PMC5345878; doi:10.1371/journal.pgen.1006633)
Supplement: S2 Table — (PDF) [file pgen.1006633.s011.pdf]

Supporting Table 2

Histone proteins that co-immunoprecipitate with H2A.B.3 identified by Mass Spectrometry as described in Table 1.

ACCESSION ID	PROTEIN NAME	GENE NAME	UNIQUE PEPTIDES #	MW (KDA)
P70696	Histone H2B type 1-A	Th2b	3	14.2
P62806	Histone H4	Hist1h4a	6	11.4
P43275	Histone H1.1	Hist1h1a	6	21.8
P27661	Histone H2AX	H2afx	8	15.1
Q07133	Histone H1t	Hist1h1t	2	21.5
F8WI35	Histone H3	H3f3a	10	15.2
Q9QZQ8	Histone macro-H2A.1	H2afy	4	39.3
P02301	Histone H3.3C	H3f3c	4	15.3

We note that the same histone proteins were also identified in H2A.Z immunopurified chromatin.
