# Supplementary material for: A new link between transcriptional initiation and pre-mRNA splicing: The RNA binding histone variant H2A.B
Source: PLoS Genet. 2017 Feb 24;13(2):e1006633. doi: 10.1371/journal.pgen.1006633 (PMC5345878; doi:10.1371/journal.pgen.1006633)
Supplement: S3 Table — (PDF) [file pgen.1006633.s012.pdf]

Supporting Table 3

Real-time PCR primer sequences for ChIP assays used in the study

ChIP primers

Ctnnd2 (exon)	Fw	5'- ACCCCCTCTGCCTTCTCTCT -3'
	Rev	5'- GCCACTGCACACACTACACG -3'
Ctnnd2 (intron)	Fw	5'- GCTCATGGATGCAGTCCCCA -3'
	Rev	5'- CGGACTGCCTAGCCCTGTTG -3'
Mpped1 (exon)	Fw	5'- GGTCTGACAGGTGCGTGCTT -3'
	Rev	5'- GTCAGGAGTTCCGGGGTGTG -3'
Mpped1 (intron)	Fw	5'- CAGTGTAGGGGGTTGCCTGC -3'
	Rev	5'- CACTCAGGATGCCCCAAGCC -3'
Cntn1 (exon)	Fw	5'- AGGTTCGAGCACACAGCGAC -3'
	Rev	5'- CGCTGATGTTGTGAACGCCG -3'
Cntn1 (intron)	Fw	5'- ACCTTGGTCCAGGCAAGCAC -3'
	Rev	5'- CCCTCCAGGGTAGGCACTGT -3'
Pkib (exon)	Fw	5'- TGGTCCCGTTGATCCTTGGACT -3'
	Rev	5'- AGGCCAGTCTCTCCCTAGCCA -3'
Pkib (intron)	Fw	5'- GTGTGAGCCGCTTCCTTCCC -3'
	Rev	5'- GAGACAGCAAATGTGCCTCGGT -3'
Tbata (exon)	Fw	5'- ACACCCAGTGCCTACCGCTT -3'
	Rev	5'- CCGCGGCCGTACCTTGGATA -3'
Tbata (intron)	Fw	5'- CTCCCCCACCTCCTGGCAAA -3'
	Rev	5'- AGTCCTGCACGCTGGGATGT -3'
Slain2 (exon)	Fw	5'- AGTCCAAATGCCAGTAGCCCATACA -3'
	Rev	5'- AGCTGCTTGACTATGGGAGGTCG -3'
Slain2 (intron)	Fw	5'- TGTGCTTGTGTGCACCACTTTGA -3'
	Rev	5'- AGAGAAAACAAGCCACCAGCAAACA -3'
Akap4 (site 1)	Fw	5'- TCAGAGCCAGCAGGAAACAG -3'
	Rev	5' - CCCTTTAGATGGAAGGGGGC -3'
Akap4 (site 2)	Fw	5'- GAGGCGTGTGCAAGGTAGAT -3'
	Rev	5'- TGGAGAAGCCACATACCAGC -3'
Akap4 (site 3)	Fw	5'- GCAATCAGAGGTCAGTTGCCA -3'
	Rev	5'- TTACGGGCCATTTGGATTACCA -3'
Il2rg (site 1)	Fw	5'- CCATGGTGCCAACAGGGATA -3'
	Rev	5'- GTGCCTGGCATCCATGTCTT -3'
Il2rg (site 2)	Fw	5'- TACCGTTTCAGCTCATCCACA -3'
	Rev	5'-AACGATCTATCCCTCACCCTTCT -3'
Il2rg (site 3)	Fw	5'- CCCTTTCCCCATAGCCTTCC -3'
	Rev	5'- GTCCTCATGTCCAGTGCGAA -3'
Akap14 (site 1)	Fw	5'-TATGCTTGGTCTCAGTAATGTCC -3'
	Rev	5' - CCTTCCTCTACAGACCAGGAGAA -3'
Akap14 (site 2)	Fw	5'- AGATGTAAGAGACATCAACAGGCA -3'
	Rev	5'- TGGGAGGATTAAGCATCGGC -3'
Akap14 (site 3)	Fw	5'- AAGAGCTTACGGCAGGTTTG -3'
	Rev	5'- ACTATATCTACCGGGTGCGCT -3'
