# Supplementary material for: A new link between transcriptional initiation and pre-mRNA splicing: The RNA binding histone variant H2A.B
Source: PLoS Genet. 2017 Feb 24;13(2):e1006633. doi: 10.1371/journal.pgen.1006633 (PMC5345878; doi:10.1371/journal.pgen.1006633)
Supplement: S4 Table — (PDF) [file pgen.1006633.s013.pdf]

Supporting Table 4

List of Illumina libraries used in this study.

Library name	mapped reads
Input Testes rep. 1 SE	17,176,324
Input Testes rep. 2 PE	195,910,428
Input Hippocampus rep.1 SE	24,659,750
Input Hippocampus rep. 2 PE	211,063,819
H2A.B.3 Testes rep. 1 SE	32,031,375
H2A.B.3 Testes tech. rep. 2 SE	272,041,885
H2A.B.3 Testes rep. 2 PE	209,303,490
H2A.B.3 Hippocampus rep. 1 SE	22,155,937
H2A.B.3 Hippocampus rep. 2 PE	186,891,694
H2A.Z Testes rep.1 SE	21,089,223
H2A.Z Testes tech. rep.2 SE	230,377'188
H2A.Z Testes rep.2 PE	186,242,182
H2A.Z Hippocampus rep. 1 SE	23,660,845
H2A.Z Hippocampus rep. 2 PE	202,335,704
H3K36me3 Testes rep. 1 PE	97,347,851
H3K36me3 Testes rep. 2 PE	95,028,143
H3K36me3 Hippocampus rep. 1 PE	115,576,309
H3K36me3 Hippocampus rep. 2 PE	82,283,392
H2A.B.3 RNA-IP 1 PE	11,406,861
H2A.B.3 RNA-IP 2 PE	22,866,609
H2A.B.3 RNA-IP 3 PE	16,496,632
H2A.Z RNA-IP 1 PE	25,818,308
H2A.Z RNA-IP 2 PE	15,572,918
H2A.Z RNA-IP 3 PE	26,365,755
Hippocampus RNA-Seq rep.1 PE	44,257,598
Hippocampus RNA-Seq rep.2 PE	49,592,421
Hippocampus RNA-Seq rep.3 PE	42,205,383
Testes RNA-Seq rep1. PE	50,206,148
Testes RNA-Seq rep2. PE	48,749,538
Testes RNA-Seq rep3. PE	51,093,859

SE-Single End Reads, PE-Paired End Reads
